# Interventions to Improve Adherence to Clinical Guidelines for the Management and Follow-Up of Pulmonary Nodules: A Systematic Review

**DOI:** 10.1016/j.chest.2025.02.031

**Published:** 2025-03-11

**Authors:** Justin Aunger, Kay Por Yip, Kamen Dosanjh, Katie Scandrett, Bianca Ungureanu, Michael Newnham, Alice M. Turner

**Affiliations:** a Department of Applied Health Research, School of Health Sciences, College of Medicine and Health, University of Birmingham, Birmingham, England; b NIHR Midlands Patient Safety Research Collaboration, University of Birmingham, Birmingham, England

**Keywords:** guideline adherence, incidental, interventions, lung cancer, pulmonary nodules, prevention, surveillance

## Abstract

**Background:**

Lung cancer is the leading cause of global cancer mortality. It is also the third most common cancer in the United Kingdom and the most prevalent worldwide. Pulmonary nodules can indicate early-stage lung cancer, but adherence to guidelines for radiologic surveillance is suboptimal, which affects early detection and treatment. Although interventions have been developed to improve follow-up, it remains unclear which approaches are most effective.

**Research Question:**

Which interventions have been developed for improving adherence to guidelines for the management of pulmonary nodules and/or the follow-up of patients, and how effective are they?

**Study Design and Methods:**

A systematic review was conducted by searching the Ovid MEDLINE, Cochrane, and Embase databases in March 2024. Reports were included of interventions of all designs that measured outcomes, including follow-up completion, guideline adherence, or early diagnosis of lung cancer. Studies relating to diagnosis, reporting screening programs, or not in English were excluded. Screening and data extraction were performed independently. Risk of bias was assessed by using three measures depending on study design.

**Results:**

A total of 3,664 titles and abstracts, including 31 studies, were identified. Six intervention types were identified: tracking systems, process improvement approaches, natural language processing systems, radiologist reporting templates, clinical decision-making support tools, and patient involvement improvements. All studies reported being effective. Tracking systems and clinical decision support tools showed significant improvements in follow-up, guideline adherence, and early cancer detection. Tracking systems may have the most potential for effectiveness because they modify more of the care pathway and use automation, reducing human error. Risk of bias was serious or critical in most nonrandomized studies.

**Interpretation:**

Our results show that there was significant variation in achieved follow-up rates across interventions; however, tracking systems seemed most effective in improving patient follow-up. Review limitations included high risk of bias and heterogeneity of included studies. Future evaluations should include more comprehensive outcome measures and rigorous designs.

## Graphical Abstract

**Figure:**
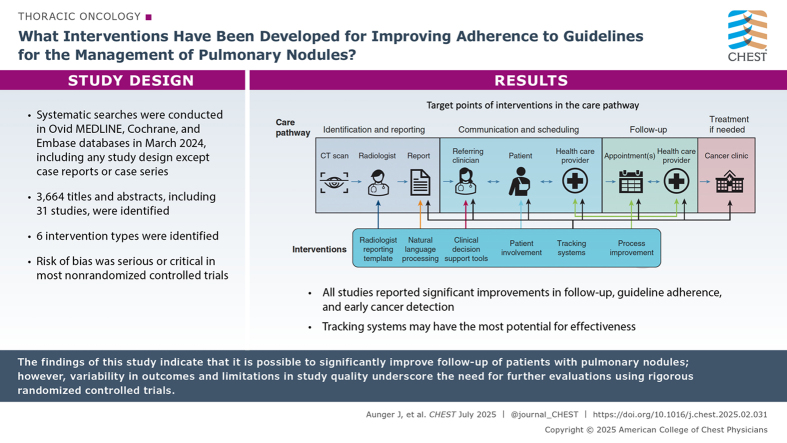

**Take-Home Points**

**Study Question:** Which interventions have been developed for improving adherence to guidelines for the management of pulmonary nodules (PNs) and/or the follow-up of patients, and how effective are they?

**Results:** Tracking systems may be the most effective intervention, by modifying more parts of the care pathway and reducing risk of human error through automated patient tracking. However, most included studies exhibited a high risk of bias.

**Interpretation:** Our results show that it is possible to significantly improve follow-up of patients with PNs; however, variability in outcomes and limitations in study quality underscore the need for further evaluations using rigorous randomized controlled trial designs. All studies were conducted in the Americas, indicating need for further research in other contexts.

Lung cancer is the third most common cancer in the United Kingdom (50,000 people per year), and has the highest international cancer mortality.^1^ Lung cancer survival has only modestly improved over time, and long-term survival remains poor (10% 10-year survival rate) with a key driver of mortality being late-stage presentation. Early-stage lung cancer is potentially curable with either surgery or radiotherapy. Thus, lung cancer screening programs have been introduced in multiple countries that aim to identify and treat disease at earlier stages.^2^ In the United States, this approach is beginning to show promising results, with annual declines in the lung cancer death rate accelerating from 3% to 5% in men and 2% to 4% in women since 2013-2014.^3^

Most early-stage lung cancers are radiographically identified as a pulmonary nodule (PNs). PNs are well or poorly circumscribed small, rounded lung abnormalities that may be single or multiple. Many PNs are identified incidentally when CT scans are performed of the chest or thorax during routine medical care, and other PNs are identified in screening programs (eg, for lung cancer). In some settings, incidental findings may be a source of more identified early lung cancers than screening programs.^2^ PNs can denote premalignant or early-stage lung cancer; therefore, effective management can reduce mortality.^4^ Up to 35% of the general population can have PNs when they undergo CT scanning, and 50% of patients with nodules have more than one.^5^

According to clinical guidelines,^5^^,^^6^ patients with nodules should be followed up systematically depending on the number of nodules, their morphology, and medical history. Although most nodules are benign, 3% to 5% turn out to be lung cancer.^5^ Therefore, in line with clinical guidelines (eg, from the British Thoracic Society and the Fleischner Society^5^^,^^6^), management involves performing follow-up scans to determine the rate of growth of these nodules. The initial follow-up scan helps determine if further surveillance is required based on growth rate of the nodule. Following up incidentally identified PNs is also important for maximizing early identification of lung cancer, as these populations can be substantially demographically different from lung cancer screening populations.^2^^,^^7^ For example, one US prospective observational study following up 22,886 patients, comparing a lung cancer screening program with a lung nodule follow-up program, found that only 54% of total patients with lung cancer would have been eligible for lung cancer screening.^2^

Evidence from around the world suggests that PNs are often followed up improperly even across very different health systems and contexts. For example, a retrospective observational cohort study in a large US academic medical center drew on the medical records of 314 patients with PNs to identify the extent of improper follow-up and whether it leads to patient harm.^8^ In this study, 14.3% of nodules turned out to be malignant, and the mean pretest probability of cancer across all patients was 11.8%. Nodule management deviated from an evidence-based strategy in 22% of patients. Among patients who received nonguideline-adherent care, 14.5% received a delayed diagnosis of cancer, a problem absent in the guideline-compliant group (*P* < .001).^8^ Other reviews in the literature have reported guideline-adherent follow-up rates as low as 29% to 39%.^9^ As such, improvements to PN follow-up processes are needed, because failure to follow up patients properly can be a failure to avoid preventable death due to lung cancer.

Suboptimal follow-up of patients can occur for many reasons, including improper comparison of findings against guidelines, errors in scheduling of follow-up, communication issues between care teams, or patient-side inability to attend appointments.^9^ There is evidence that adherence to guidelines and patient follow-up can be improved through various multifaceted interventions.^10^, ^11^, ^12^ However, to our knowledge, no comprehensive systematic review of these interventions and their effectiveness has yet been performed.

The goal of the current systematic review was to investigate as follows: (1) What interventions have been developed to improve adherence to guidelines for the management of PNs and/or the follow-up of patients? (2) How can the care pathway for PNs be improved, and which point(s) of intervention might be most effective? (3) What is the effectiveness of these interventions for improving adherence to guidelines and reducing patient harm (eg, earlier diagnosis of cancer)?

## Study Design and Methods

This systematic review was reported following the guidance of the 2020 Preferred Reporting Items for Systematic Reviews and Meta-Analyses statement (e-File 1).^13^ This study was prospectively registered with International Prospective Register of Systematic Reviews (PROSPERO) with ID CRD42024534874.

### Eligibility Criteria

#### Inclusion Criteria

##### Population

Interventions could target both staff and patients to improve the care pathway. As such, we included patients identified with PNs or health care workers managing such patients (if targeted by an intervention [eg, radiologist reporting templates]) in any care setting.

##### Study Design

Any primary study of any study design, including retrospective studies, before-and-after studies, prospective studies, and randomized controlled trials (RCTs) of various designs (eg, cluster RCTs), was eligible. This did not include other systematic reviews, case reports, or case series.

##### Intervention

Because this study sought to include a range of intervention types, study populations could be patients, health care workers, or CT scan reports.

Interventions could take place with any kind of health care provider and include any care type (eg, primary, secondary, acute, emergency). No exclusions were made based on setting. Intervention papers had to explicitly refer to either improving adherence to guidelines or improving follow-up of nodules more generally through any means. Interventions could also include means to improve follow-up recommendations made by radiologists. Clinical guidelines included any that were reputable and published (eg, Fleischner Society, British Thoracic Society).^14^ We included studies that sought to improve adherence to multiple incidental findings (eg, adrenal) if they also included PNs.

Comparators included organizations, units, or staff groups who did not receive the intervention before quality improvement initiatives took place. Comparators could also include groups of patients who received one type of care pathway for nodules vs another. Studies without comparators were summarized in e-Table 1 but not included in the synthesis.

##### Outcomes

Pathway-related outcomes including changes in patient follow-up, clinical guideline adherence, improved reporting of follow-up recommendations, impact on patient health outcomes (eg, cancer diagnosis), and economic costs/impacts of interventions.

#### Exclusion Criteria

Exclusion criteria included studies reporting interventions to improve diagnosis or detection of nodules, reporting the introduction of lung cancer screening programs but not reporting improvements to assist follow-up of nodules, studies prior to 2000 due to little use of CT scanning prior to this time, and papers not in English.

### Information Sources

Databases, including Ovid MEDLINE, Embase, and the Cochrane Central Register of Controlled Trials, were searched on March 25, 2024, to locate relevant literature. A structured Google Scholar search was also conducted on June 27, 2024, using Harzing’s Publish or Perish 8 software (available at https://harzing.com/resources/publish-or-perish), encompassing the first 200 entries. This cutoff of 200 entries was chosen to ensure only inclusion of the most relevant results.

### Search Strategy

A search strategy was iteratively developed in MEDLINE prior to translation to Embase and Cochrane. The full search strategy is depicted in e-File 2.

### Selection Process

All results were screened by 2 independent screeners at all stages (J. A., K. P. Y., K. D., and B. U.).

Rayyan.ai software (a web and mobile app for systematic reviews, available at https://rayyan.ai) was used at the title and abstract stage to facilitate screening between reviewers (no automated artificial intelligence features were used). We piloted the first 10% of titles and abstracts to check congruence and familiarity with criteria between screeners prior to proceeding.

Covidence (Veritas Health Information) was used at the full-text screening stage. Following screening at either stage, discrepancies were discussed and resolved through discussion. A third screener (M. N. or A. M. T.) was available to make final judgments if the first 2 could not agree.

### Data Collection, Data Items, and Effect Measures

Data were extracted independently in duplicate by 2 experienced reviewers (J. A. and K. P. Y.).

Data extracted from included studies were study name, authors, aims and/or objectives, country, health care setting, study design, intervention details, outcome measures, population, sample size, results summary, statistical significance, limitations summary, outcomes for each study group (with effect measures), and funding source(s). We extracted all items from included studies relating to the aforementioned outcomes, which comprised the following effect measures: completion of patient follow-up (as proportions and ORs), early diagnosis of lung cancer (ORs), time to work up results post-initial PN detection, guideline adherence by medical professionals to clinical recommendations (as proportion), completeness of radiology reports (as proportions and ORs), and positive and negative predictive value, sensitivity, and specificity of automated natural language processing (NLP) systems.

### Synthesis Methods

The included interventions were categorized according to how they intended to modify the clinical pathway. Categorization was performed inductively and independently by 2 classifiers.

Relative benefits were computed for studies in all cases in which proportions of participants broken down by outcome and study group were reported, and where interventions were relatively homogeneous (in the same category). Relative benefits were then used to generate forest plots using the metan function of StataSE version 18 (StataCorp).

Data were also synthesized narratively according to guidance by Popay et al^15^ for effectiveness studies. The narrative synthesis included developing a theory, creating a preliminary synthesis, exploring relationships within and between studies, and assessing the robustness of the synthesis. Because many heterogeneous interventions were included, we had to develop a higher-level theory to understand where interventions were attempting to improve the care pathway. Factors such as risk of bias, sample size, and quality of statistical reporting were used to assess robustness of the synthesis.

### Quality Assessment

Study risk of bias assessment was tailored to study design and conducted on the 31 studies with relevant outcomes.

The Cochrane Risk of Bias 2 (RoB 2) tool for randomized controlled trials was used for RCTs.^16^ The Cochrane Risk of Bias in Non-Randomised Studies of Interventions (ROBINS-I) tool was used to assess risk of bias in individual nonrandomized studies.^17^ A modified Prediction Model Study Risk of Bias Assessment Tool (PROBAST) tool was used for NLP studies.^18^ PROBAST signalling questions 3.3, 4.2, 4.5, and 4.9 were not assessed in this study because they are not applicable to NLP, in line with other reviews of NLP algorithms.^19^

### Reporting Bias Assessment

We did not formally assess the risk of reporting bias due to insufficient studies reporting the same outcome.

### Certainty Assessment

We did not formally assess certainty of the evidence.

### Institutional Review Board Approval

Ethical review was not required for this study as it was a review of the literature.

## Results

### Study Selection

**Figure 1:**
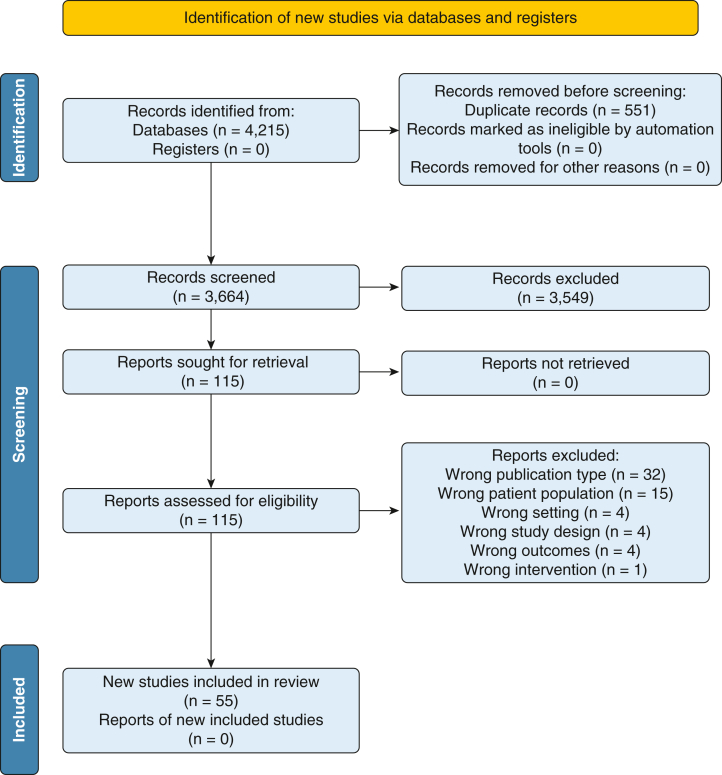
Preferred Reporting Items for Systematic Reviews and Meta-Analyses flow diagram.

We identified 3,664 unique studies, of which 3,549 were excluded. Sixty of 115 full-text items were excluded (Fig 1), resulting in 55 included studies. Of these, 31 reported outcomes of interest and were included in the synthesis.^10^, ^11^, ^12^^,^^20^, ^21^, ^22^, ^23^, ^24^, ^25^, ^26^, ^27^, ^28^, ^29^, ^30^, ^31^, ^32^, ^33^, ^34^, ^35^, ^36^, ^37^, ^38^, ^39^, ^40^, ^41^, ^42^, ^43^, ^44^, ^45^, ^46^, ^47^ Another 24 were descriptive in nature. These descriptive studies are summarized in e-File 3 and are not included in the formal synthesis.

### Study Characteristics

Table 1 summarizes the range of interventions across all 55 identified studies. e-File 3 details full characteristics of studies with effectiveness outcomes (31 of 55 included works) and the characteristics of descriptive studies.

**Table 1:** Interventions Across Included Studies and Descriptions

Intervention Type	Intervention Description	Studies Included in Synthesis
Tracking systems	Systems that are designed to keep track of patients through multiple points of the care pathway, usually from the point of a nodule being identified through to the point of follow-up being concluded in line with clinical guidelines. It can also combine multiple other intervention types (eg, a reporting template plus process improvement approach). Can use technological and/or human-based enhancements	10 studies: 11, 20, 26, 31, 33, 34, 36, 37, 39, and 47
Process improvement	Nontechnological enhancements targeting improvements in communication between radiologists, clinicians, and scheduling teams	3 studies: 28, 35, and 46
Automated NLP systems	Algorithms being developed to automatically read and classify radiology reports against clinical guidelines	7 (related to algorithm development) studies: 21, 30, 32, 38, 41, 43, and 44
Radiologist reporting templates	Templates intended to improve the reporting of all necessary information about pulmonary nodules, to ensure clinicians can properly classify these against clinical guidelines	6 studies: 10, 22, 25, 29, 42, and 45
Point-of-care clinical decision-making support tools	Systems at the respiratory physician/clinician level provide information about clinical guidelines for the management of nodules at the point of decision-making	4 studies: 23, 24, 27, and 40
Report tagging systems	Systems that add a tag to the electronic patient record and radiology reports to improve future trackability of patients	0
Patient involvement only	Interventions seeking to involve the patient more in coordinating their pulmonary nodule care	1 study: 12
National nodule registries	National databases recording patients with nodules and the status of their follow-up	0
Total		31

NLP = natural language processing.

Included intervention types were tracking systems (n = 18), process improvement (n = 11), NLP systems (n = 8), radiologist reporting templates (n = 7), clinical decision support tools (n = 6), reporting tagging systems (n = 3), patient involvement systems (n = 1), and national nodule registries (n = 1) (Table 1).

Studies were most commonly of retrospective cohort design (n = 20),^11^^,^^20^, ^21^, ^22^, ^23^^,^^25^^,^^26^^,^^28^^,^^29^^,^^31^^,^^32^^,^^34^^,^^35^^,^^37^^,^^39^^,^^41^, ^42^, ^43^^,^^45^^,^^46^ followed by pre-post (n = 5),^10^^,^^24^^,^^27^^,^^36^^,^^44^ single-masked RCT (n = 2),^40^^,^^47^ RCT (n = 1),^12^ quality improvement (n = 1),^38^ quasi-experimental stepped wedge cluster (n = 1),^33^ and comparative observational designs (n = 1).^30^ Studies were predominantly conducted in the United States (n = 29), Brazil (n = 1), and Canada (n = 1).

### Risk of Bias in Studies

#### ROBINS-I Tool

**Figure 2:**
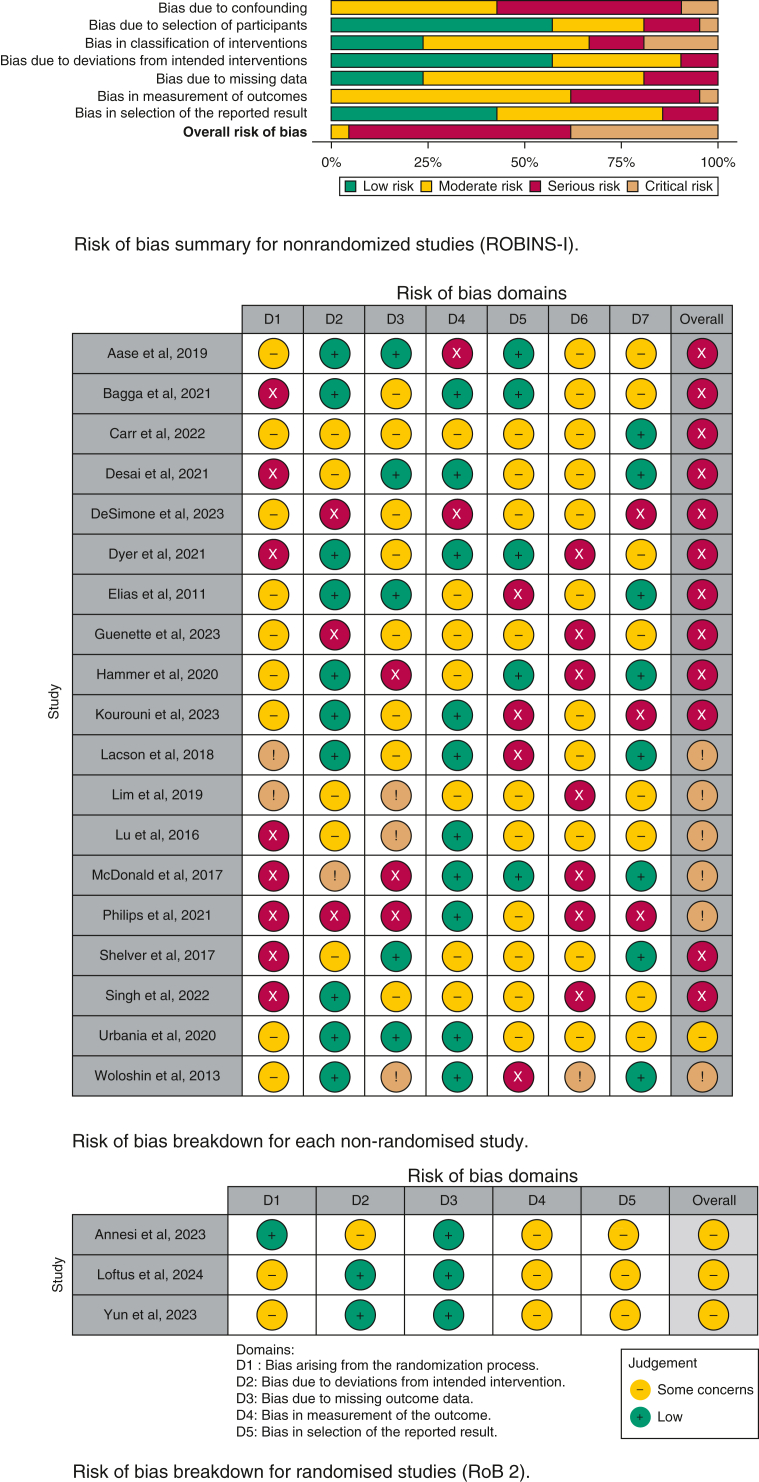
Risk of bias summary and breakdown (ROBINS-I and RoB 2). RoB 2 = Risk of Bias 2; ROBINS-I = Risk of Bias in Non-Randomised Studies of Interventions.

Twenty-one nonrandomized studies were assessed with the ROBINS-I tool.^10^^,^^11^^,^^20^^,^^22^, ^23^, ^24^, ^25^, ^26^, ^27^, ^28^, ^29^^,^^31^^,^^33^, ^34^, ^35^, ^36^, ^37^^,^^39^^,^^42^^,^^45^^,^^46^ Studies were mostly at serious (n = 12) or critical (n = 8) risk of bias (Fig 2). One further study was at moderate risk of bias. Across assessed domains, studies were most at risk due to confounding.

#### RoB 2 Tool

Three RCT studies were assessed by using the RoB 2 tool.^12^^,^^40^^,^^47^ All were found to be of “some concern,” with most bias in measurement of outcome and selection of reported results due to all not reporting preregistered statistical plans (Fig 2).

#### Risk of Bias in Algorithm Development and Validation Studies

With PROBAST, studies were found to be at high risk of bias (n = 2)^32^^,^^44^ and low risk of bias (n = 5).^21^^,^^30^^,^^38^^,^^41^^,^^43^

### Results of Individual Studies

Studies most frequently reported the proportion or odds of patients completing follow-up (n = 11)^11^^,^^12^^,^^20^^,^^26^^,^^27^^,^^31^^,^^34^^,^^36^^,^^39^^,^^46^^,^^47^; others reported guideline adherence (of decisions by medical professionals) (n = 5),^22^, ^23^, ^24^, ^25^^,^^40^ report completeness (by radiologists) (n = 4),^10^^,^^29^^,^^42^^,^^45^ time to work up results following initial nodule findings (n = 3),^28^^,^^35^^,^^46^ odds of diagnosing cancer early (n = 2),^33^^,^^34^ and malignancy rate within 24 months (n = 1).^36^ All studies reported positive results that favored the intervention groups. Table 2 summarizes the results of each study according to intervention type. Automated NLP studies are reported in e-File 3.

**Table 2:** Outcomes of Individual Studies

Study	Population	Study Design (Comparator)^a^	Intervention Description (and Areas in Pathway Targeted)	Participants	Outcome Measure (and Definition and Guidelines Used Where Available)	Results (95% CI or Proportions and Study Group)
Tracking system interventions						
Bagga et al^36^	Adults aged ≥ 35 y with chest CT scan and nodules	Pre-post (usual care)	Structured Fleischner Society recommendations and electronic tracking. • Radiologists: structured Fleischner Society reporting of PNs. • Providers: electronic tracking built on reporting system to “close loop”.	1,301 patients (882 needing follow-up); 145 (after), 737 (prior to)	• Proportion: proper follow-up (Fleischner Society criteria) • Malignancy rate within 24 mo	• 75% (553 of 737 intervention) vs 57% (82 of 145 control); *P* < .001 • 0.5% (5 of 1,046 intervention) vs 8.2% (21 of 255 control); *P* < .001
Carr et al^37^	Adults aged ≥ 18 y, Colorado residents, with nodules	Retrospective cohort (use of tracker system vs no use of tracker system)	PN tracking system that matches nodule characteristics to Fleischner Society guidelines based on tracker macros. • Radiologists: tracker phrase added to reports based on recommendation • Providers: automated system tracks if scans are > 30 d overdue. Sends automatic letters to providers • Patients: sends automatic notifications to patients	159 patients (after), 155 (prior to)	• Adjusted OR (adjusted for adenocarcinoma, nodule diameter), early-stage lung cancer	• 1.94 (1.70-3.34); *P* = .016
Desai et al^11^	Patients with nodules	Retrospective cohort (usual care)	Electronic communication tool and safety net team. • Radiologists: reporting template • Providers: automatically populated electronic options for clinician to make 1-click decisions from • Providers: human-based safety net team to monitor follow-up plans (3 FTE staff)	108 patients (after), 110 (prior to)	• Proportion: proper follow-up (defined as execution of collaborative follow-up care plans in the recommended time frame [within ± 30 d])	• 84% (91 of 108 intervention) vs 65% (71 of 110 control); *P* < .001
Dyer et al^34^	Adults aged ≥ 18 y with nodules	Retrospective cohort (preimplementation of tracker system)	Tracker phrases based on Fleischner Society guidelines together with a computerized lung nodule registry • Radiologists: template for reporting, and tracker phrases added to reports based on recommendations • Providers: backend database of PN patients Automated reminders of > 30 past due scans • Patients: automated alerts of > 30-d late scans	626 patients (after), 410 patients (prior to)	• Adjusted OR (adjusted for high risk of lung cancer, age ≥ 65 y), proper follow-up • Proportion: proper follow-up (percentage only, defined as patients receiving a subsequent report within 13 mo of the initial report)	• 1.41 (1.09, 1.81; *P* = .008) • 55% (intervention) vs 46% (control)
Elias et al^20^	Adults aged ≥ 35 y	Retrospective cohort (preimplementation of tracker system)	Standardized template for follow-up recommendations based on Fleischner Society guidelines and an electronic messaging system to notify clinicians of incidental PNs found on CT scans • Radiologists: standardized template for reporting • Providers: automated electronic alert sent to clinicians	72 patients (after), 17 patients (prior to)	• Proportion: proper follow-up (Fleischner Society guidelines ±1 mo)	• 68% (49 of 72 intervention) vs 35% (6 of 17 control); *P* = .18
Lim et al^31^	Patients with nodules	Retrospective cohort (preimplementation of tracker system)	NLP system to identify overdue cases and a custom application to query for patients with lung nodules, associated follow-up indicators, and follow-up due date. Automatic gathering of radiology reports with database to track letter sent • Providers: backend natural language processing system that identified late patients based on CT scan reports • Providers: automation of report follow-up due dates. Recording of new or changing nodules. Automatic generation of letters	5,194 patients (after), 888 patients (prior to)	• Proportion: proper follow-up (completion of subsequent scan following initial scan)	• 37% (1,942 of 5,194 intervention) vs 21% (186 of 888 control); no significance testing
Shelver et al^26^	Patients with nodules	Retrospective cohort (preimplementation of tracker system)	Automated registry that tracks nodules according to guidelines • Radiologist: use of a code when nodule identified • Providers: tracking registry, creating automatic schedule. Automated notification of overdue scans	100 patients (after), 100 patients (prior to)	• Proportion: proper follow-up (Fleischner Society guidelines ±1 mo)	• 90% (90 of 100 intervention) vs 25% (26 of 100 control); *P* < .001
Singh et al^39^	Patients who underwent CT scans	Retrospective cohort (preimplementation of tracker system)	The Nodule Net program implemented a centralized system to track lung nodules detected in chest CT scans • Radiologist: generated message to alert findings to nodule nurse navigator • Providers: nurse navigator who oversees PN tracking. Nurse holds Excel PN tracking database. Manual notification of providers of overdue scans by nurse	632 patients (after), 1,202 (prior to)	• Risk ratio: proper follow-up (Fleischner Society guidelines) • Proportion: proper follow-up (Fleischner Society guidelines)	• 1.49 (1.24, 1.79); *P* < .05 • 74% (465 of 632 intervention) vs 37% (422 of 1,202 control)
Urbania et al^33^	Adults aged ≥ 18 y who underwent CT scans	Quasi-experimental stepped wedge cluster (nonuse of tracker system)	Tagging system for radiologists plus an automated referral and follow-up system that forwards reports tagged as suspicious to a multidisciplinary team for review. A care coordinator monitored these cases and ensured they were reviewed by this team. Also integrated with electronic medical record • Radiologist: standardized reporting (optional) of PNs and tagging system of recommendations into 8 categories • Providers: a care coordinator automatically receives tagged cases • Providers: tagged cases are discussed at a multidisciplinary team meeting	1,105 (intervention), 1,751 patients (control)	• Adjusted OR (adjusted for each 6-mo time period of the study, patient age, sex, race/ethnicity, Charlson Comorbidity Index, history of cancer (other than lung or nonmelanoma skin cancer), smoking status, and radiology center clustering) for early-stage lung cancer	• 1.24 (1.09-1.41); *P* < .05
Yun et al^47^	Patients with non-mammographic outpatient diagnostic radiology reports	Single-masked RCT (single SMS reminder to patient)	An NLP algorithm and a tracking and reminder system to enhance follow-up imaging compliance • Providers: NLP system to identify patients needing follow-up • Providers: electronic organization of follow-up recommendations • Patients: automatic reminders to patients of overdue appointments	159 patients (intervention), 116 patients (control)	• Proportion: proper follow-up (American College of Radiology guidelines)	• 70% (111 of 159 intervention) vs 54% (63 of 116 control); *P* = .008
Process improvement interventions						
Kourouni et al^46^	Patients aged ≥ 18 y with electronic referral for nodules	Retrospective cohort (preintervention)	Facilitated care coordination through a dedicated nurse coordinator • Provider: dedicated nurse coordinator and database to facilitate provider-provider referrals	913 patients (after), 902 (prior to)	• Proportion: proper follow-up (appointment within 30 d of referral) • Time to workup (biopsy)	• 70% (660 of 913 intervention) vs 28% (526 of 902 control); *P* < .01 • 47.33 d (intervention) vs 79.83 d (control); *P* < .01
Phillips et al^35^	Patients with suspicious lung finding, deemed high risk for treatment delay	Retrospective cohort (routine referral)	A multidisciplinary care model aimed at improving lung cancer diagnosis and treatment for high-risk patients. It is led by a thoracic-trained advanced practice provider and involves coordinated care with oncologists and surgeons • Provider: a “clinical strategist” oversees and orders necessary treatment, coordinating with multidisciplinary team	78 patients (after), 41 (prior to)	• Time to workup	• 3 d (intervention) vs 26 d (control); *P* < .001
Wrightson et al^28^	Patients with nodules	Retrospective cohort (preintervention)	A direct radiology consult system and lung nodule evaluation team. • Provider: dedicated multidisciplinary team to facilitate workup	877 patients (prior to), 996 patients (after)	• Time to workup	• 3.4 d (intervention) vs 55 d (control); no significance testing
Clinical decision support tools						
Annesi et al^40^	Physicians, nurse practitioners, and physician assistants working in internal medicine and family medicine	Single-masked RCT (no access to guidelines)	Access to concise clinical guidelines at the point of care	44 staff (intervention), 41 (control)	• Proportion: guideline adherence (Fleischner Society)	• 96% (42 of 44 intervention) vs 71% (29 of 41 control); *P* = .003
Lacson et al^27^	Patient with PNs with findings reported in Alert Notification of Critical Results system	Pre-post (preimplementation)	Web-based systems integrated into the hospital's clinical information systems, facilitating facilitated structured documentation of key patient information, including critical findings and follow-up recommendations	327 patients (intervention), 321 (control)	• Proportion: proper follow-up (Fleischner Society guidelines)	• 26.9% (88 of 327 intervention) vs 18% 59 of 321 control); *P* < 0.01)
Lu et al^23^	Consecutive patients who had their first abdominal CT scan and a solid, noncalcified PN or mass identified on that CT scan	Retrospective cohort (before tool and concurrently without use of tool)	An integrated, point-of-care electronic system provided standardized recommendations based on the size of the nodules and patient history, such as smoking or malignancy	141 patients (intervention), 268 (control)	• Proportion: guideline adherence (modified Fleischner Society criteria, modifications included recommending immediate CT scan for nodules > 8 mm or known malignancy and patients aged < 35 y, and advising a 3-mo follow-up for high-risk patients with 7-8 mm nodules)	• 65% (92 of 141 intervention) vs 50% (133 of 268 control); *P* = .01
Zygmont et al^24^	Patients with incidental findings on consecutive CT and ultrasonographic examinations	Pre-post (preimplementation)	An education session and three primary resources: pre-created PowerScribe 360 macros, a 16-page guideline book available at workstations, and an electronic version of the guideline book with a hyperlinked table of contents	499 patients (intervention), 514 (control)	• Proportion: guideline adherence (Fleischner Society guidelines)	• 80% (400 of 499 intervention) vs 68% 347 of 514 control); *P* < .0001)
Radiologist reporting template						
Aase et al^10^	Sequential radiology reports of patients with a PN present	Pre-post (preimplementation)	Standardized dictation template covering 6 key nodule descriptors	400 reports (after), 400 reports (prior to)	• OR: completeness (Fleischner Society guidelines)	• 8.4 (5.6-12.7); *P* < .001
DeSimone et al^42^	All thoracic imaging reports by 8 radiologists	Retrospective cohort (preimplementation)	An information technology tool integrated into the radiology workflow requiring documentation of the rationale, time frame, and imaging modality for each actionable finding	56,722 reports (after), 51,323 reports (prior to)	• OR: completeness (recommendation for additional imaging containing rationale, time frame, and imaging modality)	• 1.1 (1-1.2); *P* = .03
Guenette et al^45^	Reports from consecutive adult patients who had radiology examinations	Retrospective cohort (preimplementation)	Closed-loop communication system called ARRC requiring radiologists to use structured entries for additional imaging recommendations	336 reports (after), 336 reports (prior to)	• Proportion: completed reports (explicitly includes time frame for imaging, modality, and reason)	• 46% (postintervention) vs 14% (preintervention); *P* < .001
Hammer et al^29^	Radiology reports used in alerts in intervention system	Retrospective cohort (usual care/preimplementation)	A closed-loop communication system for managing incidental PNs (this study evaluated the radiologic component)	106 (after), 77 (prior to)	• Proportion: completed reports (specification of both imaging modality and time frame)	• 71% (55 of 77 preintervention) vs 100% (106 of 106 postintervention); *P* < .001
McDonald et al^25^	Patients aged > 35 y diagnosed with a PN	Retrospective cohort (concurrent / no use of template)	The intervention involved adding a template based on the Fleischner Society guidelines to chest CT reports at the discretion of the interpreting radiologist	276 (after), 234 (prior to)	• Proportion: following guidelines (Fleischner Society guidelines)	• 45% (125 of 276 intervention) vs 31% (73 of 234 control); *P* = .0014
Woloshin et al^22^	Clinicians who treated adult patients and practiced in fields in which they might encounter an incidental PN	Retrospective cohort (standard template vs enhanced template)	Enhanced radiology report, including estimates of the probability of malignancy and specific management recommendations based on professional guidelines, compared with a standard report that only described nodule size and location	517 clinicians (all saw both conditions)	• Proportion: following guidelines (Fleischner Society guidelines)	• 72% (35%-45%) (intervention) vs 32% (control)
Patient involvement						
Loftus et al^12^	Patients with actionable incidental findings requiring follow-up	RCT (no additional notifications)	The intervention involved early direct notification to patients with actionable incidental findings requiring follow-up. It used 3 modes to do so (letter, telephone, and online portal)	593 (letter), 701 (portal), 637 (telephone), 617 (comparator)	• Proportion: completing follow-up (defined by backstop system ± 1 mo or closure via clinical care [eg, biopsy])	• 53% (328 of 617 comparator) vs 60% (385 of 637 telephone) vs 36% (255 of 701 portal) vs 59% (348 of 593 letter); *P* < .0001

ARRC = Addressing Radiologist Recommendations Collaboratively; NLP = natural language processing; PN = pulmonary nodule; RCT = randomized controlled trial.

a Study Design column includes details of study comparator group in parentheses.

**Figure 3:**
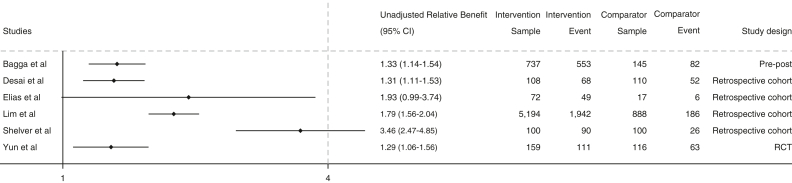
Tracking systems: relative benefit of completing follow-up in the intervention group vs usual care. RCT = randomized controlled trial.

**Figure 4:**
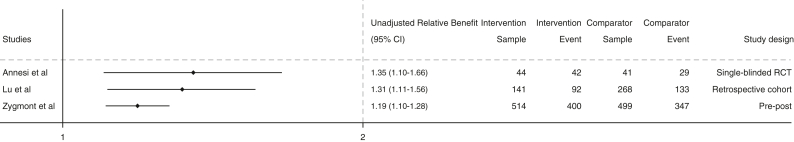
Clinical decision-making support tool relative benefit for guideline adherence vs no access to the clinical decision-making support tool. RCT = randomized controlled trial.

Computing unadjusted relative benefit was possible for tracking systems (Fig 3) and clinical decision support tool interventions (Fig 4). These results show relative benefit favoring the intervention groups across these 2 types.

### Results of Syntheses

#### Developing a Theory

**Figure 5:**
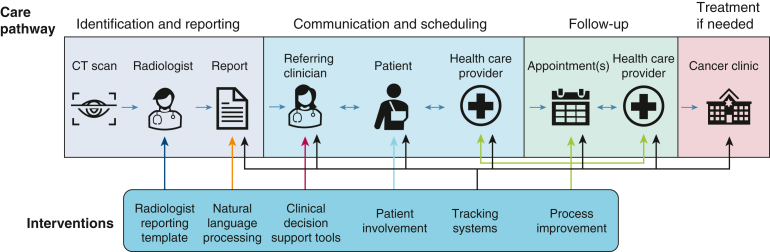
Target points of interventions in the care pathway. Arrows in color indicate the points of the lung cancer pathway that each intervention targets. The graphic shows that tracking systems (black arrow) aim to change the pathway most, with process improvement systems (green) making the second-highest number of alterations.

The first component of establishing an overview of these interventions is to set out how they attempt to affect the care pathway. Figure 5 depicts a high-level conceptual model of how interventions change particular parts of the PN care pathway. We found that tracking systems seek to alter the most points on the care pathway, often drawing in aspects of other intervention categories (eg, combining NLP algorithms and process improvement approaches). However, not all tracking systems target the entire care pathway. Table 2 shows where each tracking system targets its effects. For example, 3 sent automated reminders to patients,^34^^,^^37^^,^^47^ whereas other tracking systems only reminded clinicians or other care providers. All tracking systems needed some means for tracking patients through their care journey. Most relied on radiologists manually adding tags to the CT scan report, which often served to populate databases or registries, whereas 2 tracking systems used NLP to identify patients with nodules automatically.^31^^,^^47^

These differences highlight that there was significant variation in design of interventions even within the categories we created. As a further example, there was also variation within radiology reporting templates, as templates could vary from those reporting only guideline-required items,^10^^,^^22^^,^^25^ through to additional clinical information and specific imaging recommendations.^29^^,^^42^^,^^45^ e-File 3 fully describes these interventions. Given this heterogeneity, it was not possible to make definitive claims about intervention effectiveness, nor to synthesize these studies quantitatively.

### Developing a Preliminary Synthesis

All intervention types and studies reported being effective at improving their primary outcomes (Table 2). Of the 11 studies reporting improvements to follow-up, 8 of these were tracking systems, making cross-intervention comparisons of effectiveness for follow-up difficult. Tracking systems reported the highest relative benefits, with unadjusted relative benefits ranging from 1.29 (1.06-1.56)^47^ to 3.46 (2.47-4.85).^26^ Similarly, included tracking system studies suggest improvements in early-stage cancer diagnosis in intervention groups, with ORs of 1.24 (95% CI, 1.09-1.41)^33^ and 1.94 (95% CI, 1.70-3.34).^37^

Clinical decision support tools sought to help clinicians make decisions for follow-up that adhered to clinical guidelines (eg, Fleischner Society). These improved guideline-adherent decision-making, with increases in guideline adherence ranging from 9%^27^ to 25%.^40^ Radiology reporting template interventions sought to improve reporting, by radiologists, of all of the nodule descriptors needed to make guideline-adherent decisions. Template interventions improved report completeness (which can aid subsequent clinician decisions) by 14%^25^ to 40%.^22^ Lastly, the patient involvement intervention sought to test which notification methods about follow-up appointments led to improved follow-up completion.^12^ These authors tested 3 interventions (letter, telephone, and electronic portal) against a control and found that telephone notification of patients was most effective at ensuring follow-up completion (60%; *P* < .001), whereas an online portal was least effective (36%).^12^

All NLP algorithm interventions sought to read CT scan reports to help identify patients with nodules and/or automatically recommend decisions for clinicians. Most reported sensitivity > 90% and specificity > 80%^21^^,^^30^^,^^41^^,^^43^ except for 2.^38^^,^^44^ One study tested the efficacy of Generative Pre-trained Transformer (GPT)-3.5 and (GPT-4) large language models by OpenAI (rather than a purpose-built algorithm) for making proper clinical recommendations based on nodule characteristics.^44^ GPT-4 had low sensitivity (0.75) and specificity (0.55) and was deemed unsuitable for clinical use.

### Exploring Relationships in the Data

Tracking systems could be further subdivided into those that check and automate reminders when follow-ups are overdue^26^^,^^31^^,^^47^ and those that do not.^11^^,^^20^^,^^36^ Studies that automatically alerted clinicians about missed follow-up may be more effective than those that did not (2.18 vs 1.52 mean unadjusted relative benefit). Reporting templates showed similar strengths of effect across intervention designs.

Included interventions suggest that tracking systems can reach higher rates of follow-up completion of up to 90%, results not seen in other study types. The high rate may be because tracking systems target and improve more parts of the care pathway, mitigating more points of failure (Fig 5). However, this finding may also be due to differences in how “follow-up completion” was defined between studies (Table 2). For example, several tracking system studies used adherence to the Fleischner Society guidelines for determining follow-up completion,^20^^,^^26^^,^^31^^,^^34^^,^^36^ whereas others used completion as defined by the tracking systems themselves.^11^^,^^39^^,^^47^

Differing definitions of follow-up completion may have also affected comparator group completion rates. Across all studies, there were large differences in rates of follow-up completion in comparator groups. For example, tracking systems reported comparator group follow-up completion rates of 21%^31^ to 65%.^11^ Some studies, such as Lim et al,^31^ did not achieve postintervention follow-up completion rates equivalent to baseline or comparator group performance in other studies.

### Assessing the Robustness of the Synthesis

Overall, due to the high risk of bias across almost all included non-RCT studies, the results of this synthesis must be interpreted with caution. Furthermore, sample sizes across included studies were often small. No studies published a priori statistical analysis plans, and few reported how sample sizes were determined (eg, via sample size calculations).^12^^,^^20^^,^^34^^,^^36^^,^^42^^,^^45^

For more rigorous RCT studies, the 3 included RCT studies reported being effective. Annesi et al^40^ reported effectiveness in improving the frequency of safe answers (ie, answers that are minimally concordant with Fleischner Society guidelines but include overtreatment) with clinical decision support tools when primary care providers were presented with the clinical guidelines at the point of decision-making. However, safe answers were still only chosen in 29.3% of cases vs 4.5% preintervention (*P* = .003).

Loftus et al^12^ tested 3 different modes of early patient notification about actionable incidental findings, including lung nodules. They found that telephone calls were the only intervention significantly effective at improving completion of a 1-month follow-up scan at 60.4% (*P* < .0001) vs letters at 58.7% and an electronic portal at 36.4% (regular care, 53.2%). However, the electronic portal was most positively received by patients.

Yun et al^47^ was the only tracking system evaluated with a single-masked RCT. It found an improvement in follow-up compliance with American College of Radiology guidelines from 54% to 70% (*P* = .008), showing the promise of tracking systems.

## Discussion

This is the first systematic review to investigate strategies for improving the follow-up and adherence to clinical guidelines for PNs, and it complements the literature on wider incidental findings that require surveillance.^48^ This review is timely because there is an increasing focus on identifying lung cancer early to improve survivability, and because several different approaches have now been evaluated for tackling this patient safety issue.

We identified 6 evaluated types of intervention: process improvement approaches, radiologist reporting templates, patient involvement improvements, clinical decision support tools, NLP systems, and tracking systems. We found that tracking systems may, in theory, present the widest safety net for ensuring patient follow-up. This is likely because tracking systems address multiple parts of the pathway simultaneously, improving communication between health care providers and often sending automated reminders to reduce human error and ensure that there is a closed loop system.^34^^,^^37^^,^^47^ However, it is important to note that interventions within these types could still be different and were tested with different study designs. For example, tracking systems could vary in technological sophistication, from Excel spreadsheets manually managed by 1 person^39^ through to databases that interface with the electronic health record while using NLP to automatically track patients.^31^ This has significant implications for the extent to which these differing systems can reduce human error. Two other intervention types—report tagging systems and national nodule registries—were identified in descriptive studies only (e-File 3). Across all studies, the evidence was highly heterogeneous due to different intervention designs, study populations, and sample sizes.

We also found considerable variation in usual care performance. It is conceivable that the lower the initial rate of follow-up completion, the greater the possibility of performance improvement.^9^ Equally, no intervention managed to achieve “perfect” patient follow-up. This may partly be because improvements could still be made, and partly because some patients will inevitably not attend recommended appointments for myriad reasons. Therefore, it is not clear what the theoretical maximum successful follow-up completion rate may be. However, evidence of health inequalities in follow-up for PNs suggests that patient-related factors may be modifiable; for example, higher patient education level has been shown to significantly predict likelihood of follow-up completion in the United States.^49^ In addition, Black patients are less likely to complete follow-up than White patients.^50^ To reduce these health inequalities, future interventions should make a concerted effort to reach patients most at risk of being lost to follow-up and report patient follow-up according to sociodemographic characteristics.

A scoping review has been published that looked at “incidentalomas” more widely and included 15 studies. It found 4 main types of interventions: enhanced radiology templates, physical or verbal guideline references, electronic guideline references, and restructured clinical and communication pathways.^48^ Recommendations included advocating for more studies that incorporate outcome measures across the care pathway; for example, to show that follow-up completion has occurred, appropriate communication between patients and providers took place, and differences in early cancer diagnosis were measured. Our review was also affected by differing definitions of “follow-up completion,” with some studies, for example, using strictly interpreted Fleischner Society guidelines and others adopting these guidelines with 30 days of leeway. The move to assessing health-related outcomes is encouraging, with more recent studies recording differences in cancer diagnosis between study groups.^33^^,^^37^ However, current data are still insufficient regarding the acceptability and economic feasibility of these programs. It may make most economic sense for organizations to seek to address follow-up of multiple incidental findings (eg, adrenal and pulmonary nodules) simultaneously, as some included studies did.

Limitations of this review include uncertain results due to high risk of bias and small numbers of studies in certain intervention groups, as well as heterogeneity in populations, outcomes, and intervention designs. We originally planned to perform a meta-analysis (as per our PROSPERO protocol); however, included studies were too heterogeneous in study and intervention designs as well as outcome definitions. Unfortunately, it was also not possible to formally assess whether reporting bias was present, although the fact that all studies reported positive outcomes raises this as a possibility. Strengths included judicious use of independent screening and data extraction and inclusion of outcome measures such as completeness of follow-up and diagnosis of early-stage lung cancer, which are direct measures of relevance to patients and the safety and quality of their care.

Future studies should be planned that draw on cluster RCT designs or similar to assess effectiveness in ensuring follow-up completion and early diagnosis of lung cancer, while incorporating economic analyses and analysis of patient inequities in follow-up. We found that almost all studies were conducted in the United States, in a health system which differs significantly from the publicly funded or social insurance models present in many European countries. Thus, more implementations and robust evaluations of these systems are needed, particularly in other health systems where issues with follow-up have been similarly noted.^9^^,^^48^

## Interpretation

Several types of interventions seek to improve follow-up of patients with incidentally identified PNs, including process improvement approaches, radiologist reporting templates, patient involvement improvements, clinical decision support tools, NLP systems, and tracking systems. Although the evidence is at high risk of bias, and often of nonrandomized design, we found that tracking systems showed the most potential for improving follow-up due to targeting multiple points of the care pathway. Those that most reduce the chance of human error by automating proper closure of the follow-up loop may be particularly effective. Some tracking systems achieved up to 90% effectiveness for follow-up completion. Further research in multiple health systems, with a health inequality focus, is now essential to improving ability to diagnose lung cancer early at a treatable stage, thereby reducing unnecessary patient harm.

## Funding/Support

This study was funded by the 10.13039/501100000272National Institute for Health and Care Research Midlands Patient Safety Research Collaboration.

## Financial/Nonfinancial Disclosures

The authors have reported to *CHEST* the following: A. M. T. and J. A. received financial support for this work from the National Institute for Health and Care Research. M. N. has received other grant funding from the National Institute for Health and Care Research. A. M. T. also declares grant funding from GSK, Chiesi, CSL Behring, Grifols, and Takeda; speaking and lecture fees as well as travel reimbursement from Takeda; consulting or advisory roles and speaking fees from 10.13039/100004325AstraZeneca and AiRNA; and board memberships with Beam, Korrobio, and the Alpha-1 Foundation. None declared (K. P. Y., K. D., K. S., B. U.).

## Supplementary Data

e-Online Data

e-Online Data

e-Online Data

Audio
