# Supplementary material for: Interventions to Improve Adherence to Clinical Guidelines for the Management and Follow-Up of Pulmonary Nodules: A Systematic Review
Source: Chest. 2025 Mar 11;168(1):248–68. doi: 10.1016/j.chest.2025.02.031 (PMC12264345; doi:10.1016/j.chest.2025.02.031)
Supplement: e-Online Data [file mmc2.docx]

MEDLINE Search Strategy

| 1 | Solitary Pulmonary Nodule/ |  |
| --- | --- | --- |
| 2 | Multiple Pulmonary Nodules/ |  |
| 3 | nodul*.ti,ab. |  |
| 4 | suspic*.ti,ab. |  |
| 5 | Incidental Findings/ |  |
| 6 | Incidental imag*.ti,ab. |  |
| 7 | additional imag*.ti,ab. |  |
| 8 | incidental finding*.ti,ab. |  |
| 9 | follow-up imag*.ti,ab. |  |
| 10 | lung.mp. |  |
| 11 | Radiology/ |  |
| 12 | Radiologists/ |  |
| 13 | chest.ti,ab. |  |
| 14 | pulmonary.mp. |  |
| 15 | thorax.ti,ab. |  |
| 16 | intervention.ti,ab. |  |
| 17 | trial.ti,ab. |  |
| 18 | improve*.ti,ab. |  |
| 19 | program*.ti,ab. |  |
| 20 | tool.ti,ab. |  |
| 21 | reduc*.ti,ab. |  |
| 22 | complianc*.ti,ab. |  |
| 23 | tracking system.ti,ab. |  |
| 24 | tracking program*.ti,ab. |  |
| 25 | electronic track*.ti,ab. |  |
| 26 | Guideline Adherence/ |  |
| 27 | guideline adherence.ti,ab. |  |
| 28 | Practice Guideline/ |  |
| 29 | practice guideline*.ti,ab. |  |
| 30 | best practice*.ti,ab. |  |
| 31 | recommendation*.ti,ab. |  |
| 32 | follow-up.ti,ab. |  |
| 33 | Lost to Follow-Up/ |  |
| 34 | 10 or 11 or 12 or 13 or 14 or 15 |  |
| 35 | 26 or 27 or 28 or 29 or 30 or 31 or 32 or 33 |  |
| 36 | 1 or 2 or 3 or 4 or 5 or 6 or 7 or 8 or 9 |  |
| 37 | 16 or 17 or 18 or 19 or 20 or 21 or 22 or 23 or 24 or 25 |  |
| 38 | 34 and 35 and 36 and 37 |  |
| 39 | limit 38 to (english language and yr="2000 -Current") |  |

EMBASE search strategy

| 1 | Solitary Pulmonary Nodule/ |
| --- | --- |
| 2 | Multiple Pulmonary Nodules/ |
| 3 | nodul*.ti,ab. |
| 4 | early-stage lung cancer.ti,ab. |
| 5 | Incidental Findings/ |
| 6 | (suspicious adj1 finding*).ti,ab. |
| 7 | Incidental imag*.ti,ab. |
| 8 | additional imag*.ti,ab. |
| 9 | (incidental adj2 finding*).ti,ab. |
| 10 | follow-up imag*.ti,ab. |
| 11 | (lung adj2 (scan* or report* or nodule* or opacit*)).mp. |
| 12 | Radiology/ |
| 13 | Radiologists/ |
| 14 | (radiologic* adj2 finding*).ti,ab. |
| 15 | (chest adj2 (scan* or report*)).ti,ab. |
| 16 | (pulmonary adj2 (nodule* or opacit*)).mp. |
| 17 | (thorax adj2 (scan* or report*)).ti,ab. |
| 18 | intervention.ti,ab. |
| 19 | trial.ti,ab. |
| 20 | tool.ti,ab. |
| 21 | improve*.ti. |
| 22 | encourag*.ti. |
| 23 | complianc*.ti,ab. |
| 24 | (improve* adj2 (process or follow-up or tracking)).ti,ab. |
| 25 | ((tracking or reporting or monitoring) adj1 (system or program*)).ti,ab. |
| 26 | electronic track*.ti,ab. |
| 27 | (surveillance adj1 (program* or process)).ti,ab. |
| 28 | Guideline Adherence/ |
| 29 | guideline adherence.ti,ab. |
| 30 | Practice Guideline/ |
| 31 | practice guideline*.ti,ab. |
| 32 | best practice*.ti,ab. |
| 33 | recommendation*.ti,ab. |
| 34 | Lost to Follow-Up/ |
| 35 | Cancer diagnosis/ |
| 36 | Fleischner.ti,ab. |
| 37 | Lung-RADS.ti,ab. |
| 38 | British Thoracic Society.ti,ab. |
| 39 | 11 or 12 or 13 or 14 or 15 or 16 or 17 |
| 40 | 28 or 29 or 30 or 31 or 32 or 33 or 34 or 35 or 36 or 37 or 38 |
| 41 | 18 or 19 or 20 or 21 or 22 or 23 or 24 or 25 or 26 or 27 |
| 42 | 1 or 2 or 3 or 4 or 5 or 6 or 7 or 8 or 9 or 10 |
| 43 | 39 and 40 and 41 and 42 |
| 44 | limit 43 to yr="2000 -Current" |

Cochrane Search

1

Solitary Pulmonary Nodule/

2

Multiple Pulmonary Nodules/

3

nodul*.ti,ab.

4

early-stage lung cancer.ti,ab.

5

Incidental Findings/

6

(suspicious adj1 finding*).ti,ab.

7

Incidental imag*.ti,ab.

8

additional imag*.ti,ab.

9

(incidental adj2 finding*).ti,ab.

10

follow-up imag*.ti,ab.

11

(lung adj2 (scan* or report* or nodule* or opacit*)).mp.

12

Radiology/

13

Radiologists/

14

(radiologic* adj2 finding*).ti,ab.

15

(chest adj2 (scan* or report*)).ti,ab.

16

(pulmonary adj2 (nodule* or opacit*)).mp.

17

(thorax adj2 (scan* or report*)).ti,ab.

18

intervention.ti,ab.

19

trial.ti,ab.

20

tool.ti,ab.

21

improve*.ti.

22

encourag*.ti.

23

complianc*.ti,ab.

24

(improve* adj2 (process or follow-up or tracking)).ti,ab.

25

((tracking or reporting or monitoring) adj1 (system or program*)).ti,ab.

26

electronic track*.ti,ab.

27

(surveillance adj1 (program* or process)).ti,ab.

28

Guideline Adherence/

29

guideline adherence.ti,ab.

30

Practice Guideline/

31

practice guideline*.ti,ab.

32

best practice*.ti,ab.

33

recommendation*.ti,ab.

34

Lost to Follow-Up/

35

Cancer diagnosis/

36

Fleischner.ti,ab.

37

Lung-RADS.ti,ab.

38

British Thoracic Society.ti,ab.

39

11 or 12 or 13 or 14 or 15 or 16 or 17

40

28 or 29 or 30 or 31 or 32 or 33 or 34 or 35 or 36 or 37 or 38

41

18 or 19 or 20 or 21 or 22 or 23 or 24 or 25 or 26 or 27

42

1 or 2 or 3 or 4 or 5 or 6 or 7 or 8 or 9 or 10

43

39 and 40 and 41 and 42

44

limit 43 to yr="2000 -Current"
