# Supplementary material for: Interventions to Improve Adherence to Clinical Guidelines for the Management and Follow-Up of Pulmonary Nodules: A Systematic Review
Source: Chest. 2025 Mar 11;168(1):248–68. doi: 10.1016/j.chest.2025.02.031 (PMC12264345; doi:10.1016/j.chest.2025.02.031)
Supplement: e-Online Data [file mmc3.docx]

# Full characteristics of included studies and interventions

### Evaluation studies

| **Study** | **Country and setting** | **Study design** | **Study sample and population** | **Intervention details** | **Outcomes** | **Results summary** |
| --- | --- | --- | --- | --- | --- | --- |
| ***Tracking systems*** | | | | | | |
| Bagga et al., 2023 | USA, NYU Langone Health, a system with more than 260 outpatient office sites and 4 acute care  hospitals | Pre-post | 1301 patients with nodules | Structured Fleischner recommendations and electronic tracking | Follow-up completion pre vs. post, predictors of appropriate follow-up, malignancy diagnosis within 24m. | Delayed follow-up was reduced. |
| Carr et al., 2022 | USA, National Jewish Health Denver | Retrospective cohort | 314 patients with nodules | PN Tracking system that matches nodule characteristics to Fleischner guidelines based on tracker macros. Computerised word-finding algorithm identifies PNs and matches to providers, patient, and date of CT. Electronic registry then retrieves tracker phrases monthly to calculate expected date of follow-up and sends alert if it detects 30+ day delays. | Early stage lung cancer and associated factors (logistic regression). | The patient tracking system and computerized lung nodule registry led to an increased frequency in the diagnosis of stage 1 lung cancer. |
| Desai et al., 2021 | USA, Tertiary care referral center | Retrospective cohort | 218 patients with nodules | Electronic communication tool and safetynet team. Tool relies on radiologist-generated alerts with follow-up recommendations, then sends alerts and email notifications to PCPs via an electronic health record. PCPs can agree, disagree, or transfer the recommendation. The safetynet team ensured completion of follow-up imaging and closure of the loop. | Timely follow-up of PNs, overall follow-up of PNs, non-completion of follow-up, agreement on follow-up plan. | Improved timely follow-up for incidental pulmonary nodules from 64.5% to 84.3% and overall follow-up from 79.1% to 94.4%. Non-completion of follow-ups decreased from 20.9% to 2.8%. |
| Dyer et al., 2021 | USA, National Jewish Health - a quaternary care facility specializing in respiratory diseases | Retrospective cohort | 1036 patients >18 years with nodules | The intervention uses standardised tracker phrases based on Fleischner society guidelines incorporated into the CT reports for follow-up intervals. A computerised lung nodule registry also tracks patients to ensure timely follow-up via linking tracker phrases to the hospital EHR. It calculates due dates for follow-up scans and generated notifications for providers if follow-up imaging was overdue. | Improvement in timely follow-up, reduction in missed follow-ups, consistency in recommendations (time-based), radiologist adoption, and univariate associations of patient characteristics with follow up timing. | Proportion of patients with timely follow-up increased from 46 to 55%, proportion without follow-up decreased from 48 to 31%. Likelihood of timely follow up increased 41%. |
| Elias et al., 2011 | USA, Mayo clinic Rochester | Retrospective cohort | 89 patients with nodules | A standardized template for follow-up recommendations based on Fleischner Society guidelines and implementing an electronic messaging system to notify clinicians of incidental pulmonary nodules found on CT scans. | Total appropriate follow-up, patients at high risk with appropriate follow-up, patients at low risk with appropriate follow-up, patients with no follow up scan. | There was a nonsignificant trend towards improved time-appropriate follow-up from 35.3% (6/17) to 56.9% (49/72). |
| Lim et al., 2019 | USA, Acute care community hospital | Retrospective cohort | 9663 patients with nodules | NLP used to identify overdue cases and a custom application to query for patients with lung nodules, associated follow-up indicators, and follow-up due date. Automatic sending of notification letters with database to track letter sent. Enables presentation of clinical risk factors for clinicians also in cases where guidelines not adhered to. | Patients returning for follow up (proportion), amount overdue, follow-up completed numbers. | Increased average rate of patient follow-ups for lung nodules from 26.50 in 2015 to 59.72% in 2017. |
| Shelver et al., 2017 | USA, VA Medical Centres (9 of them) | Retrospective cohort | 200 patients with nodules | The automated lung nodule registry tracking system is linked to the VA HER and radiologists assign a unique tracker code when suspicious nodules are identified. This populates an automated registry which tracks nodules according to guidelines. The system identifies overdue scans and facilitates patient contact and rescheduling if needed, repeating until tracking is completed or cancer is identified. | The primary outcome measure was the rate of tracking failure, defined as the lack of follow-up imaging or delayed follow-up compared to guidelines. Secondary outcomes included predictors of tracking failure and reasons for tracking failure​. | Significantly reduced tracking failure from 74% to 10% across two Veterans Administration Medical Centers. Pre-implementation, the primary cause of tracking failure was loss to follow-up. Post-implementation, tracking failures were primarily due to human errors, such as radiologists not entering tracking codes or support staff template errors. Key predictors of tracking failure included younger patient age and smaller nodule size. |
| Singh et al., 2022 | USA, Mount Auburn Hospital (MAH), a 213 bed community teaching hospital in Cambridge, Massachusetts | Retrospective cohort | 632 patients who underwent CT scans | The Nodule Net program implemented a centralized system to track lung nodules detected in chest CT scans. Radiologists flagged suspicious nodules and notified a nurse navigator, who then recorded these cases in a centralized database. The nurse navigator coordinated follow-up care, contacting primary care providers or patients directly to ensure timely follow-up imaging. | The completion rate of recommended follow-up imaging for lung nodules and the detection rate of lung cancer. Reasons for incomplete follow-up and the outcomes of follow-up imaging, such as nodule stability, size changes, and malignancy diagnoses​​. | Significantly increased the completion rate of recommended follow-up imaging for lung nodules from 37% to 74%. The program facilitated timely follow-up for 78% of cases reported to it, compared to 52% without the program. The study also reported that 4% of those followed up were diagnosed with lung cancer. |
| Urbania et al., 2020 | USA, Kaiser Permanente Northern California | Quasi-experimental stepped wedge cluster design | 2856 patients who underwent CT scans | The standardised reporting and management system used a tagging system for radiologists to report their findings and the level of severity. It also has an automated referral and follow-up system which forwards reports tagged as suspicious to a multidisciplinary team for review. A care coordinator monitored these cases and ensured they were reviewed by this team. This system was integrated with the EHR. | The incidence of lung cancer diagnosis, specifically focusing on early-stage (I and II) versus late-stage (III and IV) diagnoses within 120 days of chest CT imaging. The study also assessed the time to diagnosis and the rate of surgical treatment for lung cancer within the same period. Odds of early-stage diagnosis with and without the system. | The study found that implementing a standardized reporting system for chest CT findings improved early-stage lung cancer diagnosis by 24%. However, it did not affect the diagnosis rate for late-stage cancer or increase the rate of surgical treatment within 120 days. |
| Yun et al., 2023 | USA, Einstein Medical Centre, Pennsylvania | Single blinded randomised controlled trial | 275 patients with outpatient diagnostic radiology reports | A natural language processing (NLP) algorithm and a tracking and reminder system to enhance follow-up imaging compliance. It identified patients needing follow-up based on radiology reports, organized recommendations by due date, and sent reminders via SMS. The system was designed to ensure patients were aware of and completed their recommended follow-up imaging. | The number of patients completing their recommended follow-up imaging within the American College of Radiology compliance range. | The study found that the intervention group, which received reminders through the implemented NLP algorithm and tracking system, had a significantly higher adherence rate (70%) compared to the control group (54%) for completing follow-up imaging within the ACR compliance range. |
| ***Process improvement interventions*** | | | | | | |
| Kourouni et al., 2023 | USA, Academic safety-net healthcare system with a diverse patient population in northeast Ohio encompassing a large hospital and 27 community health centers | Retrospective cohort | 1815 patients with lung nodules | The Rapid Outpatient Diagnostic Program (RODP) featured an application that automatically identified and triaged referrals for lung masses or nodules from electronic health records. It provided pulmonologists with referral and imaging data to prioritize cases by urgency. The system enabled efficient scheduling of necessary diagnostic tests and facilitated care coordination through a dedicated nurse coordinator. | The proportion of referrals completed within 30 days, racial disparities, and median time to imaging. | Significantly improved the timeliness of lung cancer referrals, with the proportion completed within 30 days rising from 48.2% to 62.4%. Notably, the program reduced racial disparities, as the completion rate for racial and ethnic minorities increased from 42.1% to 59.7%, compared to a lesser increase in White, non-Hispanic patients. The intervention also reduced the median time to PET imaging and tissue sampling, indicating more efficient diagnostic processes. |
| Phillips et al., 2021 | USA, Unclear | Retrospective cohort | 119 patients referred to thoracic surgery clinic | The Lung Cancer Strategist Program (LCSP) is a multidisciplinary care model aimed at improving lung cancer diagnosis and treatment for high-risk patients. It is led by a thoracic-trained advanced practice provider and involves coordinated care with oncologists and surgeons. The program focuses on reducing delays in diagnosis and treatment by streamlining the evaluation of suspicious lung nodules, minimizing diagnostic redundancy, and addressing disparities in care. | Time from suspicious lung finding to work up, rates of follow-up adherence, disease survival. | LCSP patients experienced shorter times from suspicious findings to work-up (3 vs. 28 days), diagnosis (30.5 vs. 48 days), and treatment (40.5 vs. 68.5 days). While the stage at diagnosis and overall survival were similar between groups, LCSP patients showed improved disease-free survival rates and better adherence to care protocols​​. |
| Wrightson et al., 2018 | USA, Robley Rex Veterans Affairs (VA) Medical Center | Retrospective cohort |  | By implementing a direct radiology consult system, the initiative aimed to reduce delays in specialist consultations, improve the detection of smaller nodules, and enhance overall healthcare efficiency. | Consultation rate, nodules identified on CT, speed of specialist review | The implementation of the Lung Nodule Evaluation Team (LNET) at the Robley Rex VA Medical Center significantly improved lung nodule management. The new system led to a notable increase in monthly consultations from 5.5 to 93, allowing for the identification of smaller nodules, with average sizes decreasing from 1.7 cm to 0.7 cm. The time from nodule identification to specialist evaluation was reduced to an average of 3.7 days. |
| ***Clinical decision support tools*** | | | | | | |
| Annesi et al., 2023 | USA, A medical centre and primary care centers | Single blinded randomised controlled trial | 85 primary care providers | Access to concise clinical guidelines at the point of care for managing the hypothetical clinical scenarios involving incidental adrenal masses (IAMs) and lung nodules. These guidelines were intended to assist the primary care providers (PCPs) in making more informed and evidence-based decisions regarding the evaluation and management of these incidental findings​​. | Safety of decisions made, guideline concordant responses | The study found that primary care providers with access to clinical guidelines made safer and more guidelines-concordant decisions, particularly in managing lower-risk incidental findings. Specifically, those with guidelines were significantly less likely to choose inappropriate management options for lower-risk adrenal masses and lung nodules compared to those without guidelines. For higher-risk scenarios, there was less difference between groups. |
| Lacson et al., 2018 | USA, 793-bed academic medical center | Pre-post | 648 inpatients and ED patients who underwent chest or abdomen CT scans | Web-based systems or modules integrated into the hospital's clinical information systems. They facilitated structured documentation of key patient information, including critical findings and follow-up recommendations. The modules integrated finalized radiology reports, allowing easy access for outpatient care providers. They improved communication of follow-up actions, especially for patients discharged from the emergency department, and replaced less efficient paper-based instructions. This initiative aimed to enhance continuity of care and reduce the likelihood of missed follow-up, thereby improving patient safety and management of pulmonary nodules​​. | The rate of follow-up management of patients with pulmonary nodules within one year after discharge, presence of documented follow-up recommendations in the discharge modules. | They significantly improved the follow-up management of patients with pulmonary nodules, increasing follow-up rates from 18% to 27%. The improvement was consistent across both inpatient and emergency department settings, with the emergency department module showing a stronger association with increased follow-up. However, despite the improvement, the overall follow-up rate remained relatively low. The study also observed that the presence of documented follow-up recommendations in the discharge modules did not significantly correlate with the actual follow-up actions taken​​. |
| Lu et al., 2016 | USA, Radiology department of an academic medical center | Retrospective cohort | 409 patients with abdominal CTs with noncalcified pulmonary nodule(s) | An integrated, point-of-care electronic system designed to improve radiologists' adherence to modified Fleischner guidelines for managing incidental pulmonary nodules detected on abdominal CT scans. It provided standardized recommendations based on the size of the nodules and patient history, such as smoking or malignancy. The tool was embedded in the radiologists' workflow, allowing them to easily incorporate guideline-based suggestions into their reports. | Clinical characteristics of patients, guideline concordance. | Guideline concordance increased from 50% to 65%. Specifically, in cases where the CDS tool was used, concordance was remarkably high at 95%, compared to 45% when it was not used. These results suggest that the CDS tool effectively standardized recommendations and improved consistency in following established guidelines​​. |
| Zygmont et al., 2016 | USA, Academic emergency radiology division consisting of 11 faculty members | Pre-post | 1013 patients undergoing consecutive CT and ultrasound exams in ED | The educational framework included a short, less than one-hour meeting to inform radiologists about the importance of adhering to societal guidelines for managing incidental findings. It provided three primary resources: pre-created PowerScribe 360 macros, a 16-page guideline book available at workstations, and an electronic version of the guideline book with a hyperlinked table of contents. | Percentage of incidental findings (IFs) managed according to societal guidelines, number managed correctly according to guidelines, and changes in management of Ifs | The intervention significantly improved compliance with societal guidelines for managing incidental findings in emergency imaging. Before the intervention, 67.5% of findings were managed correctly, which increased to 80.2% afterward. The rate of correct management for CT and ultrasonographic findings both improved, with a notable reduction in over-recommendation of additional imaging. |
| ***Radiologist reporting template*** | | | | | | |
| Aase et al., 2020 | USA, Minneapolis VA Health Care System | Pre-post | 800 patients with incidental lung nodules | The standardized dictation template developed for the study was designed to improve the reporting of incidental pulmonary nodules (PNs) by ensuring the inclusion of six key nodule descriptors such as average diameter, location, density, suspicious features, total number of nodules, and risk stratification. The template was created through a collaborative effort involving a multidisciplinary group, including pulmonary specialists, radiologists, nursing staff, and health systems personnel, based on the Fleischner Society guidelines. It was integrated into the radiology reporting system, PowerScribe 360, and was automatically presented to radiologists when interpreting scans related to PN follow-up. The implementation included training sessions and printed guides to familiarize radiologists with the template, aiming to standardize the documentation of PN characteristics across the institution​​. | The proportion of radiology reports containing all six key nodule descriptors. Usage rate of the template in different types of scans, distinguishing between initial scans where PNs were first identified and follow-up scans. The perceptions of radiologists and pulmonary nurses regarding the feasibility and acceptability of the template. | Increased the completeness of reports from 12% to 48% and improved the inclusion of critical descriptors like nodule density and suspicious features. However, template usage was higher for follow-up scans (67%) than for initial nodule identification (8%)​. |
| Desimone et al., 2023 | USA, Academic quaternary care hospital | Retrospective cohort | 176498 thoracic imaging reports | This study included two interventions. In the first, radiologists received feedback on their performance. The radiologists received periodic feedback reports that included their own RAI rates, the anonymized rates of their divisional colleagues, and the median rate for the division. These reports aimed to increase awareness among radiologists of their own practices relative to others, with the intention of encouraging more consistent and evidence-based recommendations.  The second intervention introduced an information technology (IT) tool into the radiology workflow. The tool required radiologists to document the rationale, time frame, and imaging modality for each RAI, thus promoting detailed and complete recommendations. Additionally, the tool facilitated a closed-loop communication system, whereby the referring physician was required to explicitly agree with, modify, or reject the RAI. | Rate of recommendations for additional imaging, completeness of RAIs, changes in RAI rates | The proportion of incomplete imaging recommendations decreased from 84.0% (79 of 94) during the pre-intervention period to 48.5% (47 of 97) during the IT intervention period (P < .001). |
| Guenette et al., 2023 | USA, Large academic quaternary care center | Retrospective cohort | 1008 consecutive adult patients who had radiology examinations | A closed-loop communication system called ARRC (Addressing Radiologist Recommendations Collaboratively). It requires radiologists to use structured entries for additional imaging recommendations, specifically including the imaging modality, time frame, and rationale. This tool is integrated into the radiologists' clinical workflow, enhancing the clarity and completeness of recommendations, which are then communicated to referring clinicians via automated notifications and electronic medical records. | The completeness of recommendations for additional imaging, defined by the inclusion of specific attributes (imaging modality, time frame, and rationale) in the recommendations and completeness of recommendations provided through the communication tool compared to free-text reports. | Completeness with use of the tool increased from 14% (46 of 336) before the intervention to 46% (153 of 336) (P < .001) 1 year after intervention; completeness in the corresponding free-text report language increased from 14% (46 of 336) before the intervention to 25% (85 of 336) (P < .001) 1 year after the intervention. |
| Hammer et al., 2019 | USA, Large, urban, tertiary care academic center | Retrospective cohort | 183 critical finding alerts on RADAR system for pulmonary nodules | Closed-loop communication system embedded within radiology workflow and integrated within EHR and email and paging systems to automate notification of the referring provider. Radiologist are required to specify follow-up modality and timeframe. | Report completeness | RADAR adoption increased by 75% during the study period (40% in first 3 weeks v 70% in last 3 weeks). All RADAR alerts had explicit documentation of imaging modality and timeframe for follow up, compared to 71% for non-RADAR alerts for PNs (p<0.01). |
| McDonald et al., 2017 | USA, Large, urban, tertiary care academic center | Retrospective cohort | 510 patients where one or more solid pulmonary nodules were incidentally identified | The intervention involved adding a template based on the Fleischner Society guidelines to chest CT reports at the discretion of the interpreting radiologist. This template provided standardized follow-up recommendations for incidental pulmonary nodules, classified by nodule size and patient risk factors such as smoking history. | Rate of follow up | Template group patients were significantly more likely to receive recommended follow-up care compared to control group patients (45% vs 31%, P = .0014). Most patients whose management did not adhere to Fleischner Society guidelines did not receive a recommended follow-up chest CT (210 out of 312, 67%). |
| Woloshin et al., 2014 | USA, Dartmouth-Hitchcock Medical Center | Retrospective cohort | 1075 (447 responded) clinicians | The study introduced an "enhanced" radiology report for evaluating small, incidentally detected pulmonary nodules. This enhanced report included estimates of the probability of malignancy and specific management recommendations based on professional guidelines, compared to a standard report that only described nodule size and location. Clinicians at Dartmouth-Hitchcock Medical Center were surveyed on their management choices after reviewing both report types. | Correct choice of guideline recommended management strategy | With the enhanced report, more clinicians chose the correct management strategy (72% with enhanced versus 32% with standard report [40% difference; 95% confidence interval (CI) = 35–45%]. |
| ***Natural language processing*** | | | | | | |
| Basilio et al., 2023 | Brazil, Tertiary hospital | Retrospective cohort | 300 chest CT scans (152 cases with nodules, 148 cases without). | An NLP tool to identify incidental lung nodules (ILNs) in unstructured chest CT reports. This initiative aimed to improve the detection and management of ILNs, ensuring timely follow-up and potentially early lung cancer diagnosis. | NLP algorithm sensitivity, specificity, positive predictive value, negative predictive value | The NLP tool demonstrated high accuracy in detecting incidental lung nodules (ILNs) from unstructured chest CT reports, with an internal validation sensitivity of 100% and specificity of 95.9%. In external validation across 57 hospitals, the tool achieved a sensitivity of 98.4% and specificity of 98.6%. The tool identified 93 high-risk nodules, facilitating timely follow-up and early lung cancer diagnosis in some cases. |
| Dalal et al., 2020 | USA, Three network hospitals | Retrospective cohort | 559 radiology reports (for algorithm validation) | A system for tracking follow-up imaging recommendations using radiology reports. It comprises two subsystems: one for automatically detecting follow-up recommendations using natural language processing, and another for matching these recommendations with the most likely follow-up exams using machine learning algorithms. The system uses features like patient metadata, report text similarity, and study details to identify appropriate follow-ups. | NLP algorithm sensitivity, specificity, positive predictive value, negative predictive value | The model achieved an F-score of 0.807, comparable to inter-annotator scores of 0.853 to 0.868. Key features included matching recommended modality, anatomy, and report text similarity. The system successfully differentiated true follow-ups from non-follow-ups, demonstrating potential for integration into routine clinical practice to enhance follow-up compliance. |
| Domingo et al., 2022 | USA, Healthcare system of Northwestern Medicine | Quality improvement | >570000 radiology reports (for algorithm validation) | The system utilized advanced machine learning to automatically screen radiology reports for lung and adrenal findings requiring follow-up. Integrated with the EHR, it generated Best Practice Advisories to alert physicians. The system processed reports using word embeddings and tokenisation and was trained with annotated data for accuracy. A dedicated team ensured follow-up actions were completed, improving patient care and safety. | NLP algorithm sensitivity, specificity, positive predictive value, negative predictive value. | The NLP system screened over 570,000 radiology reports, identifying approximately 29,000 requiring follow-up, with a focus on lung and adrenal findings. The system achieved a sensitivity of 77.1%, specificity of 99.5%, and a positive predictive value of 90.3% for lung findings. It facilitated nearly 5,000 interactions with physicians and tracked over 2,400 follow-ups to completion. While effective for lung findings, adrenal findings presented challenges, leading to the removal of this pathway from the clinical workflow. The initiative significantly improved follow-up adherence and reduced missed care opportunities​​. |
| Dutta et al, 2013 | USA, Tertiary care academic hospital | Retrospective cohort | 1635 radiology reports (for algorithm validation) | An NLP algorithm to detect recommendations for additional imaging in emergency department radiology reports, focusing on incidental findings. It categorized keywords into advisory terms, study types, intervals, and reasons to accurately identify follow-up needs. Integrated into the electronic health record system, the algorithm provided automated alerts to ensure effective communication of critical recommendations, aiming to prevent missed diagnoses and ensure proper follow-up care | NLP algorithm sensitivity, specificity, positive predictive value, negative predictive value. | The study found that 4.5% of emergency department radiology reports included discharge-relevant recommendations for additional imaging. However, 51% of these recommendations were not documented in discharge instructions. The final NLP algorithm developed had 89% sensitivity and 98% specificity in detecting these recommendations. |
| Evans et al., 2023 | USA, Large integrated health system | Retrospective cohort | 282 radiology reports (for algorithm validation) | An NLP and machine learning approach using the Clinical Annotation Research Kit (CLARK) to automate the detection of incidental findings in CT reports from trauma patients discharged from the ED. The method involved using regular expressions to extract relevant phrases, which were then input into a random forest model. This aimed to increase reliability of identification of significant findings. | NLP algorithm sensitivity, specificity, positive predictive value, negative predictive value. | Strong performance in identifying incidental findings in CT reports of trauma patients discharged from the ED. The random forest model achieved an area under the curve (AUC) of 0.92, with a sensitivity of 92.2% and a specificity of 79.4%. The model effectively identified incidental findings across different emergency department types, including trauma centers and community EDs. It reduced the need for manual review of reports by two-thirds, with a false negative rate of less than 3%, enhancing the detection of potentially serious conditions​​. |
| Gamble et al., 2023 | Canada, n/a - fictitious reports used | Pre-post | 450 radiology reports (for algorithm validation) | Assessing GPT-3.5, GPT-4, and a fine-tuned GPT-3.5 model for assessing guideline adherence of radiology reports | NLP algorithm sensitivity, specificity, positive predictive value, negative predictive value. | The study found that GPT-3.5 and GPT-4 performed insufficiently for clinical practice in applying the Fleischner Society guidelines to lung nodules, with accuracy rates of 5.8% and 15%, respectively. When the guidelines were provided explicitly, accuracy improved to 42% for GPT-3.5 and 66% for GPT-4. Neither model correctly recommended PET-CT or biopsy. A fine-tuned GPT-3.5 model achieved 46% accuracy, not significantly better than the original model. |
| Kang et al., 2019 | USA, NYU Langone Health, New York | Comparative observational design | 120 fictitious radiology reports (for algorithm validation) | Used SimpleNLP to identify incidental lung nodules (ILN) in unstructured radiology reports. The intervention aimed to automate the detection and management of ILNs to support quality improvement in clinical practice. | NLP algorithm sensitivity, specificity, positive predictive value, negative predictive value, guideline concordance. | The NLP tool identified incidental lung nodules with 91.1% sensitivity and 82.2% specificity. Despite implementing a standardized reporting macro, there was no significant change in follow-up recommendations or guideline concordance between 2014 and 2017, highlighting room for improvement in reporting practices​. |
| ***Patient involvement*** | | | | | | |
| Loftus et al., 2024 | USA, A healthcare network that included various facilities such as one large urban academic hospital, one urban community hospital, five rural community hospitals, and sixteen outpatient imaging sites. | Randomised controlled trial | 2548 (431 responded) patients with actionable incidental findings (AIFs) requiring follow-up | The intervention involved early direct notification to patients with actionable incidental findings (AIFs) requiring follow-up. Patients were randomized into four groups: one receiving a letter, another a phone call, and a third an electronic portal message, in addition to the standard Backstop tracking system. The fourth group served as a control and received no additional notifications beyond the existing system. The early notification aimed to improve follow-up completion rates and was designed to complement the multistage recommendation-tracking process already in place, which included escalating communications with healthcare providers​​. | Rate of recommendation follow-up compliance and clinical closure (appropriate end of care). | The study analysed data from 2,548 randomized patients, including 593 notified by letter, 637 by phone, 701 through a portal, and 617 in the control group. Group 3 had the lowest follow-up completion rate within one month of the due date at 36.4%, compared to 58.7% for group 1, 60.4% for group 2, and 53.2% for group 4 (P < .0001 for all comparisons). Group 2 was the only group with a significantly higher completion rate than group 4 (P = .014). Patients expressed a preference for early notifications and favoured communication through the electronic portal. |

### Descriptive study characteristics

| **Study** | **Country and setting** | **Study design** | **Study sample and population** | **Intervention type and details** | **Outcomes** | **Results summary** |
| --- | --- | --- | --- | --- | --- | --- |
| (Del Gazio and Allen, 2024) | USA, unclear setting | Descriptive | n/a | Tracking system.  AI-based system. Recommendations entered by nurse navigator. NLP detects AIFs in radiology reports and correct follow-up plan is displayed to nurse navigator to populate order for clinician to review. AI then communicates to patient and provider using mail and electronic methods. System can tailor communication to those most at risk. | n/a | n/a |
| (Emani et al., 2019) | USA, emergency department | Descriptive | Patients with nodules | Tracking system.  Working groups, creation of patient reports, workflow redesign, patient tracking. A registry was also created and NLP used to identify patients in need of follow up. Outreach performed by humans/not automated. | Rate of follow-up only after intervention | 56.9% (70 out of 123 patients) received appropriate follow up |
| (Fu et al., 2023) | USA, academic medical centre in Boston | Descriptive | Emergency department attendee with incidental findings | Tracking system.  New workflow with streamlined actionable incidental findings reporting system software with automated notification of providers, PCPs, and a nurse navigator. Education of radiologists on use of the system. System uses Fleischner guidelines. Nurse navigator works to coordinate the follow-up. | Patient characteristics in system, referral rates, rates of lung nodules or other AIFs, rate of proper follow-up recommendations in system | Only 10 out of 258 reports audited did not provide proper follow-up recommendations |
| (Irani et al., 2020) | USA, Unclear setting | Descriptive | Unclear | Tracking system.  Radiologist report tagging system plus NLP. Appointment of champions to spread use of the system. QI nurses employed to check whether follow-up was performed properly on a monthly basis. Weekly huddles to find further impovements. | Patients who otherwise would have been lost (unclear how known) | 177 patients identified within 9 months who would otherwise have been lost |
| (Kang et al., 2020) | USA, large academic medical centre | Descriptive | Uses of macro | Tracking system.  Macro system which automates recommendations based on imaging findings and records in the electronic health record. System enables tracking also of communication of follow-up test completion etc. | Use of the macro | Increasing use of the macro over time |
| (Kapoor et al., 2023) | USA, Urban academic quaternary care hospital | Descriptive | Patients with incidental findings | Tracking system.  Software solution for entering follow-up recommendations. Automated sending of emails to referrer which enabled information about recommendation but did not automatically book follow-ups. Automatic checking of follow-up completion after requisite amount of time with automatic reminders. | Follow-up completion, predictors of follow-up completion (socioeconomic, inpatient, ED, care setting, referrer surgical specialty, age, race, ethnicity, language, insurance status) | 74.8% rate of completion of radiologist recommended follow-up imaging deemed clinically necessary |
| (Mahajan et al., 2023) | USA, Adult emergency departments at large community hospital | Observational | Patients 16 years and older who underwent various imaging procedures at the adult Eds | Tracking system.  A noncommercial electronic medical record (EMR)-based tool to identify incidental pulmonary nodules (IPNs) and enhance patient communication through lung navigators. | Number of IPNs identified, nodule characteristics, number of patients contacted, cancer diagnoses, follow-up | Lung navigators contacted 87% of patients, leading to 12 cancer diagnoses (2%): 5 early-stage non-small cell lung cancer, 3 late-stage and 1 extensive-stage small cell lung cancers. This approach improved follow-up rates significantly compared to the previous 31%, demonstrating the tool's effectiveness in identifying IPNs and ensuring patient communication and follow-up. |
| (Schwartz et al., 2021) | USA, Large University Medical Center | Retrospective cohort | All patients reported via unexpected findings system from Oct 2019 to Oct 2020 | Tracking system.  Closed loop communication program that employed three full-time navigators with extensive clinical experience to facilitate communication between radiologists, providers, and patients. Radiologists documented findings in a dedicated EHR tab, triggering alerts and adding the case to the navigators' worklist. The navigators ensured follow-up actions, such as scheduling further imaging or specialist referrals, and patient notifications through electronic messages, phone calls, or certified letters. | Number of examinations with unexpected findings, median time to patient notification, number of follow-up actions, types of unexpected findings, user satisfaction, communication success | The study reported 3542 unexpected findings in a year, with a median notification time of 12 days. Follow-up actions included 2127 additional imaging studies and 1078 patient referrals, primarily identifying potential neoplasms (11%). The system was well-received, with 89% of radiologists and 65% of providers finding it useful. |
| (Blodgett et al., 2023) | USA, an urban tertiary care teaching hospital, a suburban community hospital, and an urgent care centre | Observational | Patients who visited the Emergency Department (ED) or Urgent Care Center (UCC) | Process improvement.  An EHR-independent initiative involving an experienced nurse who reviewed laboratory and imaging studies from discharged ED and UCC patients. The nurse ensured appropriate follow-up for any incidental findings (IFs), coordinating with an on-call emergency physician when needed | Total number of imaging abnormalities, number of patients called back to ED, cost effectiveness | The QA program reviewed over 1.4 million tests, identifying 6,530 incidental findings, mainly from laboratory and imaging studies. The most common intervention was contacting primary care physicians, with 21 patients called back to the ED, leading to potentially life-saving interventions in 10 cases. The cost per abnormality identified was $42.48, and the cost per potentially life-saving intervention was $27,743. |
| (Jiang et al., 2023) | USA, Two EDs in NYC | Retrospective | Patients ranging from 21 to 105 years of age who were referred to the Pulmonary Nodule Program | Process improvement.  The program involved a structured workflow that included notifying patients, coordinating follow-up care, and referring to subspecialists such as pulmonologists and oncologists. | Rate of successful follow up after ED visits, number lost to follow up, number who received diagnosis of malignancy, characteristics of patients lost to follow up | The study reviewed 574 patients referred to the Pulmonary Nodule Program, finding a 69.1% initial follow-up rate. Of these, 46.8% were actively followed within the program, while 30.5% were lost to follow-up. Among those who completed follow-up, 13% were referred for biopsy, with a significant portion diagnosed with malignancy. There was minimal difference in demographic characteristics between those who followed up and those who did not. |
| (Johnson et al., 2015) | USA, Radiology department at NYU Langone Medical Centre | Retrospective | Outpatient diagnostic imaging examinations | Process improvement.  Integrated into the radiologists' dictation software, the tool allows radiologists to flag cases requiring follow-up, triggering a notification system. An administrative assistant manages the communication by faxing the report to the referring physician and confirming receipt via phone. The system documents all communication actions, ensuring reliable and efficient information transfer with minimal disruption to the radiologists' workflow, particularly for findings classified as Category 3​​. | Effectiveness and reliability of the communication tool in conveying important nonurgent imaging findings. The outcomes included the successful faxing and phone confirmation of reports, the speed of these communications, and the subsequent clinical actions taken by referring physicians. The study also measured the impact of the tool on radiologists' workflow, focusing on how well the tool facilitated communication without significant disruptions. Additionally, it assessed the frequency and nature of follow-up actions taken in response to the communicated findings​​. | The study found that the communication tool was highly effective, with over 99% of reports successfully faxed and phone confirmation received. Communication typically occurred within one business day, with many cases handled within hours. The tool effectively minimized workflow disruptions for radiologists. About 60.7% of reports included recommendations for further imaging, of which 47% were acted upon. For reports involving "in-network" providers, 41% led to documented follow-up actions, such as additional patient consultations or referrals. |
| (Mannix et al., 2021) | USA, Academic medical centre in Boston | Within-group analysis | Patients who underwent imaging studies during October and November 2016 and had nonurgent but clinically significant findings that required follow-up. | Process improvement.  A notification system for overdue radiology follow-ups at an academic medical center. Providers were notified via electronic medical records, phone calls, and pagers if follow-ups were not completed within a specified time. This system aimed to improve follow-up compliance rates for nonurgent radiology recommendations and identify additional clinically important diagnoses. | The rate of completed follow-ups for overdue radiology recommendations and the identification of additional clinically important diagnoses following the notification system's implementation. | Before the intervention, 26% of patients had incomplete follow-ups. After notifying providers, this rate decreased to 20.7%, representing a 20.4% reduction in noncompliance. Out of 680 eligible patients, 36 completed their follow-ups post-notification, leading to the identification of four clinically important diagnoses, including one biopsy-proven malignancy. |
| (Roberts et al., 2020) | USA, Massachusetts General Hospital | Retrospective | Patients who were evaluated at the Pulmonary Nodule and Lung Cancer Screening Clinic | Process improvement.  A multidisciplinary clinic for managing pulmonary nodules. The clinic, staffed by specialists from various medical fields, coordinated care for patients with pulmonary nodules. The team reviewed cases in preclinic meetings, made joint treatment recommendations, and provided comprehensive follow-up, including imaging, biopsies, and surgeries. A nurse navigator facilitated patient understanding and adherence to care plans. | Adherence rate, number of patients diagnosed with malignancies, timeliness of care, patient follow up | The study evaluated 1,136 patients at the PNLCSC, finding that 19% had pathologically confirmed or empirically treated pulmonary malignancies. The adherence rate to clinic recommendations was 95%. Most patients (95%) diagnosed with non-small cell lung cancer (NSCLC) had early-stage disease. The median time from initial appointment to intervention was less than 50 days. The multidisciplinary approach facilitated timely care, reduced unnecessary procedures, and maintained high adherence to care plans, indicating a successful model for managing pulmonary nodules. |
| (Van Gerpen, 2021) | USA, Acute care hospital | Descriptive | n/a | Process improvement.  A program to systematically identify and manage incidental pulmonary nodules found on CT scans. Led by An APRN-CNS and a multispecialty advisory group, the program aimed to improve the consistency of reporting, ensure patients and primary care providers were informed, and recommended appropriate follow-up actions. | n/a | The study identified that 26% of incidental pulmonary nodules detected required follow-up, with approximately 1,100 patients per year needing further evaluation. The program improved the consistency of reporting by using Fleischner guidelines and ensured communication between emergency department providers, trauma services, and primary care providers. The initiative also resulted in the early detection of approximately 45 cases of lung cancer, including 15 early-stage cases. |
| (Verdial et al., 2020) | USA, Seattle Cancer Care Alliance Lung Cancer Early-Detection and Prevention Clinic | Retrospective | Patients aged >35 years with an incidentally detected lung nodule evaluated in a multidisciplinary clinic | Process improvement.  The multidisciplinary nodule clinic (MDNC) involved a team of pulmonologists, thoracic surgeons, and chest radiologists. The clinic evaluated patients with incidentally detected lung nodules, utilizing the 2005 Fleischner Society Guidelines for management. The team provided a comprehensive approach to care, aiming to promote guideline-concordant evaluations while accommodating individualized care for complex cases. | Rates of guideline-concordant recommendations and care received, reasons for deviations from guidelines, and associated diagnostic outcomes, including lung cancer and benign nodules. Number of patients receiving guideline-concordant care. | The study found that 67% of patients received guideline-concordant recommendations, but only 58% received guideline-concordant care. Deviations occurred due to concerns over multiple diagnoses needing early detection or alternative provider recommendations. Among the patients, 29% were diagnosed with lung cancer, with most cases in those receiving guideline-concordant care. No lung cancer cases were found in patients receiving less intense care. |
| (Weinstock et al., 2019) | USA, Mt Auburn Hospital | Retrospective | Lung nodule patients who underwent chest computed tomography (CT) scans at Mount Auburn Hospital between January 1, 2015, and December 31, 2016. | Process improvement.  The program identified patients needing follow-up. It involved contacting providers and patients to ensure appropriate surveillance, especially when recommendations were not documented or followed. | Completion rate of recommended follow-up imaging or consultations for patients with detected lung nodules and the identification of new or growing nodules, including cases of stage 1 lung cancer. Patient outreach success and the reasons for incomplete follow-up | The study reviewed 9,224 CT scans, identifying 5,101 with lung nodules. Of these, 1,202 patients required follow-up, and outreach efforts targeted them. 801 patients had providers within the system, leading to a 27% follow-up rate, while 302 had external providers, with a 40% follow-up rate. Two patients were diagnosed with stage 1 lung cancer, and 23 had new or growing nodules. The initiative highlighted the need for a robust follow-up system, as 24% of patients initially lacked appropriate follow-up, demonstrating the program's effectiveness in enhancing surveillance​​. |
| (Cavallo et al., 2023) | USA, Emergency department of an academic radiology department | Retrospective | n/a | Automated NLP intervention.  A quality assurance program using AI and NLP to detect pulmonary nodules in emergency department CT scans. Used NLP to read radiology reports but also a semiautomated email system to alert physicians and track relevant studies. | n/a | The study processed 19,246 CT scans, identifying 50 suspected discrepancies between AI and NLP analyses. Of these, 34 required addenda, leading to 20 new follow-up imaging recommendations for patients. The majority of missed Nodules were found in abdominal and pelvic scans rather than chest-specific scans. The implementation of the quality assurance program led to improved detection of pulmonary nodules. |
| (Alkasab et al., 2017) | USA, general hospital | Descriptive | n/a | Clinical decisionmaking support tool.  An open framework designed to integrate clinical guidelines into radiology reporting. It uses a standardized, machine-readable format (XML) to encode guidelines, facilitating consistent, guideline-concordant reports. The system includes a reference implementation for testing and an open authoring environment, enabling radiologists, expert groups, and vendors to develop and refine decision support tools. The goal is to reduce variability in radiology reports, improve adherence to clinical guidelines, and enhance the quality and efficiency of patient care​​. | n/a | The study successfully developed the CAR/DS framework, which effectively encoded clinical guidelines into a standardized format for integration with radiology reporting tools. The system was validated through a reference implementation, demonstrating its ability to produce consistent, guideline-concordant reports. The open authoring environment encouraged participation from the radiology community, aiming to reduce report variability and improve adherence to clinical standards. The results indicated that the CAR/DS system could enhance the quality and efficiency of radiology reporting, potentially benefiting patient care by providing clearer and more standardized diagnostic information​​. |
| (Bizzo et al., 2021) | USA, radiology department | Descriptive | n/a | Clinical decisionmaking support tool.  The Computer-Assisted Reporting and Decision Support (CAR/DS) framework integrates clinical decision support tools into radiology workflows, promoting standardized and structured reporting. It incorporates clinical guidelines to assist radiologists in creating consistent and guideline-compliant reports, using predefined data elements and AI algorithms. CAR/DS enhances radiologist education at the point of care and facilitates the generation of structured data, useful for clinical management, trials, and AI development. | n/a | The framework may improve the standardization and quality of radiology reporting in cancer imaging by reducing variability in reports among radiologists and enhancing compliance with clinical guidelines. The integration of decision support tools within the framework may enable more accurate and consistent reporting, including appropriate follow-up recommendations. Additionally, the CAR/DS framework may support the incorporation of artificial intelligence (AI) algorithms, aiding in data analysis and decision-making. The system also provided educational benefits for radiology trainees and non-specialists, facilitating point-of-care learning through structured examples and guidelines. |
| (Tremblay et al., 2022) | Canada, Multiple hospitals and an outpatient private radiology clinic | Mixed methods | Participants undergoing low-dose computed tomography (LDCT) scans for lung cancer screening | Radiology reporting template.  The electronic synoptic reporting tool developed for LDCT lung cancer screening standardizes radiology reports by providing a structured template. It includes sections for nodule details, incidental findings, and auto-generated narrative reports. The tool also integrates nodule risk calculations and Lung-RADS classification, facilitating clear communication of findings and management recommendations. It supports automated participant result letters and allows for easy data analysis and program monitoring​​. | Frequency of omissions in reports (only pre) | The system improved report completeness and facilitated real-time nodule risk assessment. In 2,815 LDCT reports, the system reduced reporting omissions, with radiologists agreeing with 95.3% of auto-generated management plans. The system enhanced program management and communication. |
| (de Oliveira Filho et al., 2022) | Brazil, Verticalised healthcare system | Descriptive | n/a | Report tagging system.  The study implemented a standardized coding system and automated text mining tool for radiology reports to identify and notify relevant findings in imaging exams. This intervention aimed to streamline the referral process to specific care lines, reduce waiting times for consultations and treatments, and enhance patient management efficiency. The system involved coding significant findings, extracting these codes daily using Python scripts, and updating dashboards for a dedicated medical team to review and decide on appropriate actions. | n/a | Between October 2020 and September 2021, the study analyzed 40,296 CT and MR exams from 35,944 patients. Approximately 69.5% of patients had at least one relevant clinical finding. Follow-up data were available for 10,019 patients, with 33.2% referred to specialists, mainly thoracic surgery. Key findings included calcified coronary artery plaques, pulmonary nodules, and suspected liver disease. The automated system improved the efficiency of identifying and managing significant findings, with 64.7% of recommended actions completed. Some patients could not be contacted or refused care, highlighting areas for improvement. |
| (Dusendang et al., 2021) | USA, Integrated, community-based health-care system | Descriptive | n/a | Report tagging system.  The tagging system implemented at Kaiser Permanente Northern California assigns specific codes to chest CT reports to classify pulmonary findings. Codes like "#PUL5" indicate findings suspicious for malignancy, prompting automatic referrals for further review by a multidisciplinary team. Other tags, such as "#PUL6" for known lung cancer or suspected metastasis, help streamline the follow-up process. The system aims to ensure timely and accurate diagnoses, reduce care gaps, and improve the management of lung nodules. It also supports primary care providers by offering standardized follow-up recommendations based on the type and risk level of the pulmonary findings. | n/a | Out of 39,409 tagged chest CT scans, 2.8% resulted in new lung cancer diagnoses within 120 days. The "#PUL5" tag, indicating suspicious findings, had a sensitivity of 74%, specificity over 95%, and a positive predictive value of 36%. Chart reviews revealed opportunities to improve tagging accuracy and radiologist training. Combining "#PUL5" and "#PUL6" increased sensitivity to 84%. The tagging system effectively categorized patients and facilitated appropriate follow-up, but enhancements were recommended for better accuracy and management of lung nodules. |
| (Murphy et al., 2016) | USA, Large VA network of 7 hospitals and associated clinics | Descriptive | n/a | Report tagging system.  A computerized trigger algorithm to identify delays in the follow-up of abnormal chest imaging results suspicious for malignancy. This algorithm was applied to a national clinical data warehouse containing electronic health records from VA healthcare facilities. The algorithm flagged patients for potential delays, excluding cases where follow-up was unnecessary. The criteria included factors like terminal illness, completed biopsies, or hospice care. A sample of flagged cases was manually reviewed to confirm delays. The intervention aimed to ensure timely follow-up by alerting healthcare providers to potential delays, thereby improving patient care and outcomes. | n/a | The study applied the trigger algorithm to 208,633 patient records, identifying 1,847 cases flagged as suspicious for malignancy, with 655 being trigger-positive. Manual review of 400 trigger-positive cases revealed 158 confirmed delays in follow-up and 84 cases needing additional tracking. The trigger achieved a positive predictive value (PPV) of 61%, a negative predictive value (NPV) of 97%, sensitivity of 99%, and specificity of 38%. |
| (Borg et al., 2024) | Denmark, Joint Secretariat for the Danish Clinical Quality Improvement Program | Descriptive | n/a | National nodule registry.  The Danish Lung Nodule Registry is a nationwide initiative aimed at systematically recording pulmonary nodules to enhance early detection and management of lung cancer. As part of the Danish Lung Cancer Registry, it introduces new ICD-10 codes to classify nodules into solid, part-solid, and non-solid types. The registry will capture around 7000 nodules annually, focusing on patients' follow-up and clinical progression. This systematic approach aims to improve patient outcomes by ensuring appropriate follow-up for nodules, thereby enhancing early cancer diagnosis. The registry integrates with other national databases, providing comprehensive data for research and healthcare planning. | n/a | It has integration with the Danish Lung Cancer Registry, enhancing data quality and facilitating a comprehensive overview of pulmonary nodules. It identified risk factors such as smoking and occupational exposures, providing insights for public health interventions. The registry offers valuable research opportunities, aiding in understanding the epidemiology and natural history of nodules. |

# References (descriptive studies)

Alkasab, T.K., Bizzo, B.C., Berland, L.L., Nair, S., Pandharipande, P.V., Harvey, H.B., 2017. Creation of an Open Framework for Point-of-Care Computer-Assisted Reporting and Decision Support Tools for Radiologists. Journal of the American College of Radiology : JACR 14, 1184–1189. https://doi.org/10.1016/j.jacr.2017.04.031

Bizzo, B.C., Almeida, R.R., Alkasab, T.K., 2021. Computer-Assisted Reporting and Decision Support in Standardized Radiology Reporting for Cancer Imaging. JCO Clinical Cancer Informatics 5, 426–434. https://doi.org/10.1200/CCI.20.00129

Blodgett, M., Fradinho, J., Gurley, K., Burke, R., Grossman, S., 2023. The Value of Using a Quality Assurance Follow-Up Team to Address Incidental Findings After Emergency Department or Urgent Care Discharge: A Cost Analysis. Journal of Emergency Medicine 65, e568–e579. https://doi.org/10.1016/j.jemermed.2023.08.001

Borg, M., Rasmussen, T.R., Hilberg, O., 2024. Introduction of the Danish Lung Nodule Registry: A part of the Danish Lung Cancer Registry. Cancer epidemiology 89, 102543–102543. https://doi.org/10.1016/j.canep.2024.102543

Cavallo, J.J., de Oliveira Santo, I., Mezrich, J.L., Forman, H.P., 2023. Clinical Implementation of a Combined Artificial Intelligence and Natural Language Processing Quality Assurance Program for Pulmonary Nodule Detection in the Emergency Department Setting. Journal of the American College of Radiology : JACR 20, 438–445. https://doi.org/10.1016/j.jacr.2022.12.016

de Oliveira Filho, E.N., Machado, F.P., Almeida, M.F.A., Barbosa, P.N.V.P., 2022. Automated Notification of Relevant Expected or Incidental Findings in Imaging Exams in a Verticalized Healthcare System. Journal of Medical Systems 46, 55–55. https://doi.org/10.1007/s10916-022-01842-y

Del Gazio, A.J., Allen, Z., 2024. Actionable Incidental Findings on Radiologic Examinations: Existing Challenges for Nurse Navigator Led Tracking Programs and Resolving Capabilities of an Artificial Intelligence-Enabled Solution. Journal of Radiology Nursing. https://doi.org/10.1016/j.jradnu.2024.01.004

Dusendang, J.R., Sakoda, L.C., Urbania, T.H., Ely, S., Osinski, T., Patel, A., Herrinton, L.J., 2021. An Intervention to Tag Findings Suspicious for Lung Cancer on Chest Computed Tomography Has Good Sensitivity and Number Needed to Diagnose. The Permanente journal 25. https://doi.org/10.7812/TPP/20.155

Emani, S., Sequist, T.D., Lacson, R., Khorasani, R., Jajoo, K., Holtz, L., Desai, S., 2019. Ambulatory Safety Nets to Reduce Missed and Delayed Diagnoses of Cancer. Joint Commission Journal on Quality and Patient Safety 45, 552–557. https://doi.org/10.1016/j.jcjq.2019.05.010

Fu, T., Berlin, S., Gupta, A., Plecha, D., Sunshine, J., Sommer, J., 2023. Implementing a Streamlined Radiology Workflow to Close the Loop on Incidental Imaging Findings in the Emergency Department. Journal of digital imaging 36, 776–786. https://doi.org/10.1007/s10278-022-00773-x

Irani, N., Saeedipour, S., Bruno, M.A., 2020. Closing the Loop-A Pilot in Health System Improvement. Current problems in diagnostic radiology 49, 322–325. https://doi.org/10.1067/j.cpradiol.2020.02.006

Jiang, L.G., Cahill, M., Chansakul, A., Steel, P.A.D., Sullivan, D., Pua, B.B., 2023. A Collaborative Emergency Medicine and Radiology Pulmonary Nodule Program: Identification of Associated Efficacy and Outcomes. Journal of the American College of Radiology : JACR 20, 796–803. https://doi.org/10.1016/j.jacr.2023.04.020

Johnson, E., Sanger, J., Rosenkrantz, A.B., 2015. Important nonurgent imaging findings: use of a hybrid digital and administrative support tool for facilitating clinician communication. Clinical imaging 39, 493–496. https://doi.org/10.1016/j.clinimag.2015.01.002

Kang, S.K., Doshi, A.M., Recht, M.P., Lover, A.C., Kim, D.C., Moore, W., 2020. Process Improvement for Communication and Follow-up of Incidental Lung Nodules. Journal of the American College of Radiology : JACR 17, 224–230. https://doi.org/10.1016/j.jacr.2019.11.023

Kapoor, N., Lynch, E.A., Lacson, R., Flash, M.J.E., Guenette, J.P., Desai, S.P., Eappen, S., Khorasani, R., 2023. Predictors of Completion of Clinically Necessary Radiologist-Recommended Follow-Up Imaging: Assessment Using an Automated Closed-Loop Communication and Tracking Tool. American Journal of Roentgenology 220, 429–440. https://doi.org/10.2214/AJR.22.28378

Mahajan, A.K., Collar, N., Bari, M., Nader, A., Muldowney, F., Patel, P.P., Weyant, M.J., Druckenbrod, G.G., Oliverio, P., Moynihan, J., Deeken, J.F., 2023. Effectiveness of an Electronic Medical Record-Based Recognition Tool for the Identification of Incidental Pulmonary Nodules. Journal of Bronchology and Interventional Pulmonology 30, 373–378. https://doi.org/10.1097/LBR.0000000000000905

Mannix, J., LaVoye, J., Wasserman, M., Lada, N.E., Onoue, K., Hassan, K., Sarangi, R., Haroon, S., Gaffar, A., Qureshi, M.M., Gupta, A., 2021. Notification system for overdue radiology recommendations improves rates of follow-up and diagnosis. American Journal of Roentgenology 217, 515–520. https://doi.org/10.2214/AJR.20.23173

Murphy, D.R., Meyer, A.N.D., Bhise, V., Russo, E., Sittig, D.F., Wei, L., Wu, L., Singh, H., 2016. Computerized Triggers of Big Data to Detect Delays in Follow-up of Chest Imaging Results. Chest 150, 613–620. https://doi.org/10.1016/j.chest.2016.05.001

Roberts, T.J., Lennes, I.T., Hawari, S., Sequist, L.V., Park, E.R., Willers, H., Frank, A., Gaissert, H., J.-A., S., Ryan, D., 2020. Integrated, Multidisciplinary Management of Pulmonary Nodules Can Streamline Care and Improve Adherence to Recommendations. Oncologist 25, 431–437. https://doi.org/10.1634/theoncologist.2019-0519

Schwartz, F.R., Roth, C.J., Boardwine, B., Hardister, L., Thomas-Campbell, S., Lander, K., Montoya, C., Jaffe, T.A., 2021. Electronic Health Record Closed-Loop Communication Program for Unexpected Nonemergent Findings. Radiology 301, 123–130. https://doi.org/10.1148/radiol.2021210057

Tremblay, A., Ezer, N., Burrowes, P., MacGregor, J.H., Lee, A., Armstrong, G.A., Pereira, R., Bristow, M., Taylor, J.L., MacEachern, P., Taghizadeh, N., Koetzler, R., Bedard, E., 2022. Development and application of an electronic synoptic report for reporting and management of low-dose computed tomography lung cancer screening examination. BMC medical imaging 22, 111–111. https://doi.org/10.1186/s12880-022-00837-y

Van Gerpen, R., 2021. Creating an Incidental Pulmonary Nodule Safety-Net Program. Chest 159, 2477–2482. https://doi.org/10.1016/j.chest.2020.12.053

Verdial, F.C., Madtes, D.K., Cheng, G.-S., Pipavath, S., Kim, R., Hubbard, J.J., Zadworny, M., Wood, D.E., Farjah, F., 2020. Multidisciplinary Team-Based Management of Incidentally Detected Lung Nodules. Chest 157, 985–993. https://doi.org/10.1016/j.chest.2019.11.032

Weinstock, T.G., Tewari, A., Patel, H., Kelley, K., Tananbaum, R., Flores, A., Shah, A.T., Abujaber, S.Y., Khorashadi, L., Shortsleeve, M.J., Thomson, C.C., 2019. No stone unturned: Nodule Net, an intervention to reduce loss to follow-up of lung nodules. Respiratory Medicine 157, 49–51. https://doi.org/10.1016/j.rmed.2019.09.003
